# Indices of Change, Expectations, and Popularity of Biological Treatments for Major Depressive Disorder between 1988 and 2017: A Scientometric Analysis

**DOI:** 10.3390/ijerph16132255

**Published:** 2019-06-26

**Authors:** Bach X. Tran, Giang H. Ha, Giang T. Vu, Long H. Nguyen, Carl A. Latkin, Kalpana Nathan, Roger S. McIntyre, Cyrus S. Ho, Wilson W. Tam, Roger C. Ho

**Affiliations:** 1 Institute for Preventive Medicine and Public Health, Hanoi Medical University, Hanoi 100000, Vietnam; 2 Johns Hopkins Bloomberg School of Public Health, Johns Hopkins University, Baltimore, MD 21205, USA; 3 Vietnam Young Physicians’ Association, Hanoi 100000, Vietnam; 4 Institute for Global Health Innovations, Duy Tan University, Hanoi 100000, Vietnam; 5 Center of Excellence in Evidence-Based Medicine, Nguyen Tat Thanh University, Ho Chi Minh City 70000, Vietnam; 6 Center of Excellence in Behavioral Medicine, Nguyen Tat Thanh University, Ho Chi Minh City 70000, Vietnam; 7 Stanford University School of Medicine, 291 Campus Drive, Stanford, CA 94305, USA; 8 Institute of Medical Science, University of Toronto, Toronto, ON M5S 1A8, Canada; 9 Mood Disorders Psychopharmacology Unit, University Health Network, Toronto, ON M5G 2C4, Canada; 10 Department of Psychiatry, University of Toronto, Toronto, ON M5T 1R8, Canada; 11 Department of Toxicology and Pharmacology, University of Toronto, Toronto, ON M5S 1A8, Canada; 12 Department of Psychological Medicine, National University Health System, Singapore 119228, Singapore; 13 Alice Lee Centre for Nursing Studies, Yong Loo Lin School of Medicine, National University of Singapore, Singapore 119077, Singapore; 14 Center of Excellence in Evidence-Based Medicine, Nguyen Tat Thanh University, Ho Chi Minh City 70000, Vietnam; 15 Department of Psychological Medicine, Yong Loo Lin School of Medicine, National University of Singapore, Singapore 119228, Singapore; 16 Institute for Health Innovation and Technology (iHealthtech), National University of Singapore, Singapore 117599, Singapore; 17 Center of Excellence in Behavioral Medicine, Nguyen Tat Thanh University, Ho Chi Minh City 70000, Vietnam

**Keywords:** antidepressants, depressive disorder, electroconvulsive therapy, neurostimulation, scientometric analysis

## Abstract

Background. Major Depressive Disorder (MDD) is the most common psychiatric disorder with high prevalence and disease burden. Biological treatments of MDD over the last several decades include a wide range of antidepressants and neurostimulation therapies. While recent meta-analyses have explored the efficacy and tolerability of antidepressants, the changing trends of biological treatments have not been evaluated. Our study measured the indices of change, expectations, and popularity of biological treatments of MDD between 1988 and 2017. Methods. We performed a scientometric analysis to identify all relevant publications related to biological treatments of MDD from 1988 to 2017. We searched the Web of Science websites for publications from 1 January 1988 to 31 December 2017. We included publications of fluoxetine, paroxetine, citalopram, sertraline, amitriptyline, fluvoxamine, escitalopram, venlafaxine, duloxetine, milnacipran, desvenlafaxine, levomilnacipran, clomipramine, nortriptyline, bupropion, trazodone, nefazodone, mirtazapine, agomelatine, vortioxetine, vilazodone, electroconvulsive therapy (ECT), repetitive transcranial magnetic stimulation (rTMS), vagus nerve stimulation (VNS), deep brain stimulation (DBS), and transcranial direct current stimulation (tDCS). We excluded grey literature, conference proceedings, books/book chapters, and publications with low quality as well as publications not related to medicine or human health. The primary outcomes assessed were indices of change, expectations, and popularity. Results. Of 489,496 publications identified, we included 355,116 publications in this scientometric analysis. For the index of change, fluoxetine, sertraline and ECT demonstrated a positive index of change in 6 consecutive periods. Other neurostimulation therapies including rTMS, VNS, DBS and tDCS had shown a positive index of change since 1998. We calculated the index of change of popularity index (PI), which indicates that from 2013 to 2017, the number of publications on tricyclic antidepressants (TCAs) and monoamine oxidase inhibitors (MAOIs) were reduced by 85.0% and 81.3% respectively, as compared with the period 2008–2012. For the index of expectation, fluoxetine and ECT showed the highest index of expectations in six consecutive periods and remained the highest in 2013–2017. For popularity, the three antidepressants with highest PI were fluoxetine (4.01), paroxetine (2.09), and sertraline (1.66); the three antidepressants with lowest PI were desvenlafaxine (0.08), vilazodone (0.04) and levomilnacipran (0.03). Among neurostimulation therapies, ECT has the highest PI (2.55), and tDCS the lowest PI (0.14). The PI of SSRI remained the highest among all biological treatments of MDD in 2013–2017. In contrast, the PI of ECT was reduced by approximately 50% during the period 2008 to2012 than that in the period 2013 to 2017. Conclusions. This scientometric analysis represents comprehensive evidence on the popularity and change in prospects of biological treatments for MDD from 1988 to 2017. The popularity of SSRI peaked between 1998 and 2002, when their efficacy, tolerability and safety profile allowed them to replace the TCAs and MAOIs. While the newer neurostimulation therapies are gaining momentum, the popularity of ECT has sustained.

## 1. Introduction

Depression is the most common psychiatric disorder with the aggregate point, one-year and lifetime prevalence of 12.9%, 7.2%, and 10.8% respectively [1]. Based on the Diagnostic Statistical Manual-5 (DSM-5), the lifetime prevalence was 15.2% for Persistent Depressive Disorders (PDD) with persistent major depressive episode (MDE), 3.3% for PDD with pure dysthymia, 28.2% for Major Depressive Disorder (MDD), and 9.1% for Other Specified Depressive Disorders (OSDD) [2]. The World Health Organization Disability Assessment Schedule (WHO DAS II) demonstrated that depression is associated with disability in primary care setting [3]. Depressive disorder based on International Statistical Classification of Diseases and Related health Problems (ICD-10) predicted disability pension when adjusted for sex and age [4]. MDD is estimated to reach second place in the ranking of Disability Adjusted Life Years (DALY) calculated for all ages by 2020 [5]. Biological treatments including various types of antidepressants and neurostimulation therapies are the mainstay of treatment for MDD. The WHO (2018) reported that less than half of those were depressed, and in some countries less than 10% received treatment [5]. Untreated MDD has far-reaching consequences as it leads to work-related disability and productivity loss which result in adverse effects on quality of life, incurring significantly higher indirect costs to the society [6,7,8,9].

Over the last several decades, biological treatments of MDD have made significant progress. The mechanism of action for all available antidepressants is mainly based on the monoamine mechanisms [10]. Tricyclic antidepressants (TCA) were introduced in the 1950s, which proved to be efficacious but potentially fatal in overdoses. The monoamine oxidase inhibitors (MAOIs), while also effective, have serious side effects including hypertensive crisis, and are hence used rarely. The introduction of selective serotonin reuptake inhibitors (SSRIs) was instrumental in making treatment readily available because of their safety profile and tolerability. 

Recently, Cipriani et al. (2018) performed a network meta-analysis to evaluate the efficacy of 21 antidepressant drugs except for neurostimulation therapies [11]. All antidepressants were found to be more effective than placebo. Agomelatine, amitriptyline, escitalopram, mirtazapine, paroxetine, venlafaxine and vortioxetine were more effective than other antidepressants. Fluoxetine, fluvoxamine, reboxetine and trazodone were least efficacious antidepressants. Agomelatine and fluoxetine were found to be most acceptable with the least side effects. In contrast, amitriptyline, clomipramine, duloxetine, fluvoxamine, reboxetine, trazodone and venlafaxine had the most side effects. Nevertheless, this meta-analysis was mainly focused on double-blind, randomized controlled trials (RCTs) of antidepressants but not able to provide information on other clinical or animal studies and neurostimulation therapies for MDD. 

Previous scientometric analyses in psychiatry focused on illicit drug addiction [12], child psychiatry [13], bipolar disorder [14], depression and suicide [15]. The lack of understanding of the changes in publication patterns of the biological treatments of MDD and expectations of the academic community are significant gaps of knowledge in the current medical literature. Scientometric analysis utilizes the following isometrics [16]: (1) the popularity index (PI), representing the proportion of articles on a particular biological treatment, relative to all articles on the topic of MDD; (2) the index of change, representing the degree of growth in publications on a biological treatment compared to the previous period; (3) the index of expectations, representing the ratio of the number of articles on a biological treatment in the top 20 medical journals and (4) the index of change of PI, showing the change in the proportion of publication of old treatment in one period compared with that of the first 5 years of the study period (1988–1992).

Therefore, we performed a scientometric analysis to measure the index of popularity, change, and expectations of 23 antidepressants and 5 neurostimulation therapies including electroconvulsive therapy (ECT), repetitive transcranial magnetic stimulation (rTMS), transcranial direct current stimulation (tDCS), vagus nerve stimulation (VNS) and deep brain stimulation (DBS) at 5-year intervals from 1988–2017. We hypothesized that there would be no change in the index of popularity, change, and expectations of 23 antidepressants and 5 neurostimulation therapies at 5-year intervals from 1993 to 2017 as compared to baseline period 1998–1992.

## 2. Methods

### 2.1. Search Strategy and Selection Criteria

The Web of Science (WOS) was used to search scientific articles related to depression published between 1 January 1988 to 31 December 2017. We decided to choose the WOS based on the following reasons: 1) the WOS included articles published in higher quality journals compared to other databases (e.g., Google Scholar); 2) PubMed focuses on biomedical studies only and resulted in selection bias while the WOS includes articles from different fields (e.g., Psychology) [17]; 3) the WOS covers oldest scientific papers since 1900. 

The keywords “Depress*” (including depression and depressive) OR “antidepressant*” (including antidepressant and antidepressants) OR “tricyclic-antidepressant” OR “TCA”, OR “selective serotonin reuptake inhibitor” OR “SSRI”, OR “serotonin noradrenaline reuptake inhibitor” OR “SNRI”, OR “monoamine oxidase inhibitor” OR “MAOI”, OR ‘noradrenaline/norepinephrine-dopamine reuptake inhibitor” OR “NDRI” OR “noradrenergic specific serotonergic antidepressant” OR “NASSA”, OR “serotonin antagonist and reuptake inhibitor” OR “SARI”, OR “electroconvulsive therapy” OR “ECT” OR “repetitive transcranial magnetic stimulation” or “rTMS” OR “vagus nerve stimulation” OR “VNS” OR “deep brain stimulation” OR “DBS” OR transcranial direct current stimulation” OR “tDCS” were used to search all articles and reviews from 1988 to 2017 that contained these words in the title, abstract or keywords (see Supplementary Materials 1. Search strategy). We focused on documents published in peer-reviewed journals with identifiable authors [18]. Therefore, other document types such as grey literature, conference proceedings, or books/book chapters were not included in the analysis. We excluded non-English articles due to 1) the WOS covers most English journals, for non-English papers, only the titles were translated into English 2) the remarkable increase of English articles submitted by researchers in non-English speaking countries [19,20]. We only included research areas related to human health and medicine. As a result, we excluded 82 subject areas (see number 3, Supplementary Materials 1. Search strategy). 

Twenty-three antidepressants were searched in various kinds of literature: six SSRIs (fluoxetine, paroxetine, citalopram, sertraline, fluvoxamine, escitalopram); five SNRIs (venlafaxine, duloxetine, milnacipran, desvenlafaxine, levomilnacipran); three TCAs (amitriptyline, clomipramine and nortriptyline); one NDRI (bupropion); two SARIs (trazodone and nefazodone), one NaSSA (mirtazapine), and three new antidepressants (agomelatine, vortioxetine and vilazodone). We included five neurostimulation therapies: ECT, rTMS, VNS, DBS, and tDCS. The name of each antidepressant and therapy above was entered in the search box with the combination of the above keywords and “depress”. In addition to the terms related to the primary field of depression or depressive disorder, we applied the sub-specialty areas: Psychiatry, Neuroscience, Neurology, Psychology, Pharmacology and Pharmacy. These sub-specialties are based on the research areas defined by the WOS. 

### 2.2. Data Extraction

Data including the publication year, total papers published per year per biological treatment and the number of papers published per biological treatment were extracted. 

### 2.3. Outcomes and Statistical Analysis

In the evaluation of biological treatments for MDD, we applied the following indicators [16,21,22].

#### 2.3.1. Popularity Index (PI)

The PI is the share of papers on a specific topic (the name of an antidepressant or neurostimulation therapy) relative to all articles in the field of MDD in a period of time. 

#### 2.3.2. Index of Change

The index of change is the percentage change in the number of publications of an antidepressant or neurostimulation therapy during a period of five years compared to the previous five years. This index reflects the change of interest on a biological treatment for MDD. indicating that the increase in the number of publications (in percentage) on a biological treatment in the whole field of MDD during the current period as compared to previous period.

#### 2.3.3. Index of Expectations

The index of expectations or top journal selectivity index is the ratio of the number of articles of an antidepressant or neurostimulation therapy in the top 20 journals relative to the total number of articles in biological treatments for MDD. It reflects the level of interest on the particular biological treatment of MDD in the top journals. We used the WOS to select the 20 top journals for each antidepressant or therapy in consecutive five-year periods (see Supplementary Materials 2). 

With the 23 antidepressants, the criteria for selection of an antidepressant into further analysis was the level of its PI during 1988–2017. If the PI was higher than 0.5 [22], an antidepressant or a neurostimulation therapy would be further assessed using the index of change, and expectations.

### 2.4. Ethics Statement

Ethical approval was not required for this study as it does not involve direct involvement of research participants.

## 3. Results

### 3.1. Study Selection

**Figure 1:**
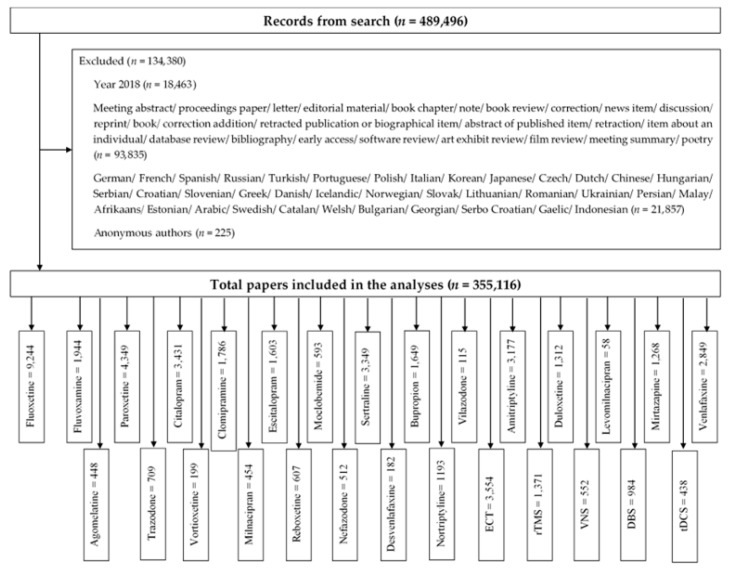
Study selection process.

The search identified a total of 489,496 publications. We excluded 134,380 publications that did not meet inclusion criteria: 1) research article or review; 2) non-English papers; 3) anonymous author (Figure 1). We included 355,116 publications in the analysis. Figure 1 summarizes the number of included studies for each biological treatment.

### 3.2. The Indices of Popularity, Change and Expectation

The PI indicates the biological treatments which were the subject of the highest number of publications for the treatment of MDD in the period 1988–2017. Of the 23 antidepressants included in the search, eight antidepressants have a PI larger than 0.5%. Table 1 summarizes the index of change and index of expectation of eight antidepressants with PI > 0.5% and five neurostimulation therapies. Six antidepressants with PI > 0.5% were SSRIs, one was an SNRI, and one was a TCA. Among the eight antidepressants with PI > 0.5%, the popularity of two SSRIs, fluoxetine and sertraline increased in the past 30 years with a positive index of change in six consecutive periods. The popularity of other SSRIs including paroxetine, citalopram and fluvoxamine showed a recent decline with a negative index of change. The popularity of TCAs including clomipramine and amitriptyline has reduced since 1998 with a negative index of change for four consecutive periods. Among the eight antidepressants, the index of change of venlafaxine showed the greatest reduction in popularity by magnitude in 2008–2012 and 2013–2017. The index of expectations tracks the prospect of a biological treatment based on the selectivity of top 20 journals. Fluoxetine showed the highest index of expectations in six consecutive periods and remained the highest in the 2013–2017 period. For neurostimulation therapies, ECT was the only neurostimulation therapy with PI > 0.5. The popularity of ECT has increased in the past 30 years with a positive index of change in six consecutive periods. The other neurostimulation therapies showed an upward trend in popularity with the positive index of changes from 1998 for rTMS, VNS and DBS and 2003 for tDCS. ECT showed the highest index of expectations in 6 consecutive periods (> 1%) and remained the highest in 2013–2017. For rTMS, the index of expectations was 1.28% in 2008–2012 and increased to 1.44% in 2013–2017. For tDCS, the index of expectations increased by less than 1% during each period. The index of expectations of DBS increased to 1.43% in 2013-2017 as compared with 0.88% in 2008–2012.

The PI of major classes of antidepressants and neurostimulation therapies in four sub-specialties are presented in Table 2. The PI of three classes of antidepressants were highest in the field of Pharmacology and Pharmacy (26.33 for SSRI, 3.33 for SNRI and 1.88 for TCA). Conversely, the PI of ECT was highest in the field of Psychiatry (2.55). The PIs of rTMS (1.04), VNS (0.23), DBS (0.56) and tDCS (0.29) were highest in the field of Neurosciences and Neurology.

The PI of 23 antidepressants and neurostimulation therapies are presented in Table 3. The three antidepressants with the highest PI were fluoxetine (4.01), paroxetine (2.09) and sertraline (1.66). The three antidepressants with lowest PI were desvenlafaxine (0.08), vilazodone (0.04) and levomilnacipran (0.03). Among neurostimulation therapies, ECT had the highest PI (2.55), and tDCS had the lowest PI (0.14).

**Figure 2:**
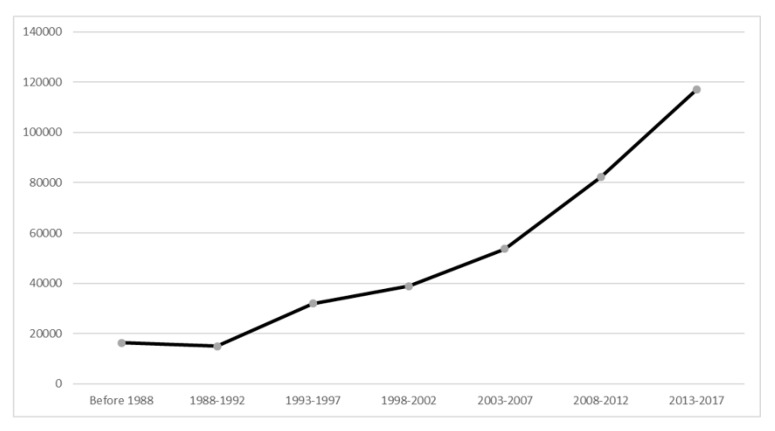
Number of publications on biological treatments for major depressive disorder from 1988 to 2017.

Over the past 30 years, there had been a steady increase in the number of publications. The number of publications in the period 2013-2017 was 117,115, accounted for 33% total papers in the field of MDD and increased by 617% as compared with the period 1988–1992 (*n* = 16,331) (see Figure 2).

**Figure 3:**
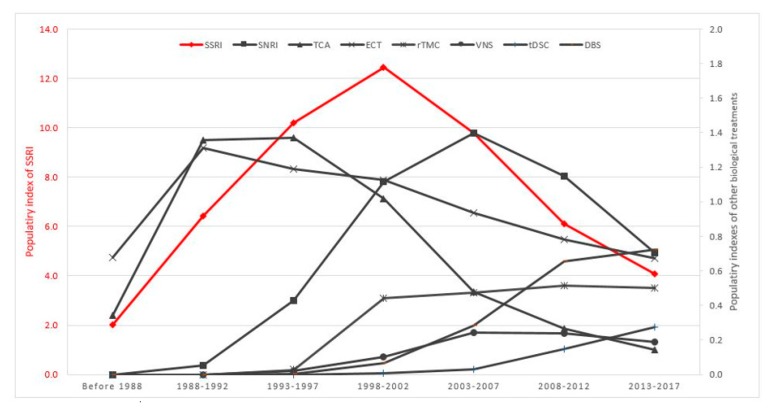
Time course of PI for biological treatments for MDD (1988–2017).

The PI for major classes of antidepressants and neurostimulation therapies for the treatment of MDD between 1988 and 2017 is shown in Figure 3. SSRI, SNRI and TCA had a PI of 4.99, 0.57 and 1.20 respectively in the first 5-year period (1988–1992). The PI of SSRI reached its peak at 12.10 in the period 1998–2002. Subsequently, the PI for SSRI was reduced by 60% from its peak during 1998–2002, to 4.98 during 2013–2017. Similarly, there was a gradual, upward trend in the PI of SNRI and TCA during the period from 1988 to 2002. However, the PI of SNRI and TCA reduced sharply in the next 15 years, about 80% in the period 2013–2017 compared with the highest PI of SNRI and TCA. In contrast, the PI of ECT remained steady and consistently higher than 0.5 during 1988–2017. The PI of three neurostimulation therapies (rTMS, tDCS and DBS) had increased continuously and gradually. The PI of SSRI remained the highest among all biological treatments of MDD in 2013–2017.

Table 4 shows the index of change for journal articles on biological treatments of MDD in psychiatry and medical journals from 1988 to 2017. The index of change of journal articles on biological treatments of MDD during the periods of 1993–1997 (65.2%) and 1998–2002 (34.3%) were higher than the index of change of those in psychiatry and medical journals. The index of change of journal articles on biological treatments of MDD started to decline from 2003 onwards. Between 2013 and 2017, the index of change of journal articles on biological treatments of MDD (12.0%) was half of the index of change of the ones in psychiatry (24.8%) and less than the index of change of those published in medical journals (22.0%). It is also of interest that the index of change for psychiatry-specific publications was always higher than that of all articles published in medical journals throughout the study period.

### 3.3. The Index of Change of PI

Table 5 presents change in the PI of current antidepressants and neurostimulation therapies as compared to older antidepressants (e.g., TCA and MAOI) and ECT. There was a strong reduction in the index of change of PI of TCA and MAOI. In 2013–2017, the PI of MAOI and TCA was reduced by 81.3% and 85.0% respectively, as compared with that of 2008–2012.

During the same period, the PI of ECT had reduced gradually from −17.5% during 1993–1997 to −48.1% during 2013–2017. The index of change of SSRI and SNRI had increased during 1993–1997 and 1998–2002. Nevertheless, the growth rate has reduced since the period 2003–2007, and index of change took a negative direction in the subsequent periods. 

## 4. Discussion

### 4.1. Principal Findings

The key findings of this scientometric review are summarized as follows: The popularity of SSRIs remained the highest among all biological treatments of MDD in 2013–2017. Three SSRIs, fluoxetine, paroxetine and sertraline were the subject of the highest number of publications. By comparison with other antidepressants, the popularity of fluoxetine and sertraline had increased in the past 30 years with a positive index of change in 6 consecutive periods. The popularity of the older antidepressants such as the TCAs had declined since 1998. For neurostimulation therapy, the popularity of ECT had increased in the past 30 years with a positive index of change in 6 consecutive periods. The PI of rTMS, VNS, DBS and tDCS were higher in the field of Neurosciences and Neurology compared to Psychiatry. Although the PI of other neurostimulation therapies including rTMS, tDCS and DBS had increased, they could not replace ECT. The popularity of SSRIs reached its peak in 1998–2002. The index of change of journal articles on the biological treatment of MDD started to decline since 2003. 

### 4.2. Possible Explanations of Findings Related to Antidepressants

Three antidepressants, fluoxetine, paroxetine and sertraline had the highest PI. The Canadian Network for Mood and Anxiety Treatments (CANMAT) guidelines recommend all SSRIs as first-line treatment with level 1 evidence (evidence from at least 1 RCT) [23]. SSRIs, in addition to tolerability and efficacy, have a safety profile which is a significant advantage over older antidepressants. Fluoxetine has the longest half-life which allows the patient to take on an alternate day, and this may enhance adherence. Besides its antidepressant effect, fluoxetine offers neuroprotection [24] and reduces the risk of medical comorbidity associated with MDD [25]. Paroxetine, which has anticholinergic properties, was initially promoted for treating anxiety associated with depression [26]. Due to a higher incidence of serotonin withdrawal syndrome [27], a controlled-release (CR) formulation of the paroxetine was developed and maintained its popularity [28]. Nevertheless, the use of paroxetine during the first trimester is associated with cardiac malformations in the foetus. As a result, paroxetine was labelled as category D for teratogenicity [29]. The popularity of sertraline had increased throughout the study period. It was found to be superior to fluoxetine in improving workplace functioning after six months of therapy [7]. Our results suggest that current antidepressants and neurostimulation therapies had been more popular than TCA and MAOI. Based on the CANMAT guidelines, TCA and MAOI are second-line antidepressants, and irreversible MAOI is the third-line antidepressant [23]. Our findings are congruent with a recent meta-analysis which found that TCA such as amitriptyline and clomipramine had the most side effects [11]. TCA and MAOI are relatively less safe compared to SSRIs. TCAs are associated with higher rates of acidosis, cardiac conduction problems, respiratory depression, and seizures [22]. MAOIs are associated with high rates of hypertension, confusion, increased creatinine, and fever [22].

Three antidepressants with lowest PI are desvenlafaxine, vilazodone and levomilnacipran. Desvenlafaxine is a synthetic form of the isolated major active metabolite of venlafaxine. Its effectiveness is comparable to the parent drug, venlafaxine which is an established SNRI. It is not surprising that desvenlafaxine does not obtain much popularity because it does not offer additional therapeutic advantages as compared to the parent drug. Furthermore, its parent drug, venlafaxine was found to be one of the antidepressants with the most side effects in a network meta-analysis [11]. Venlafaxine has been associated with conduction disturbance, tachycardia, and seizure, and has a higher mortality index [30]. Levomilnacipran and vilazodone are relatively new antidepressants, and these antidepressants are considered as second-line treatment in the CANMAT guidelines due to lack of relapse prevention data at the time of approval [31]. A recent network meta-analysis reported that the efficacy of levomilnacipram was not directly compared with at least another antidepressant [11]. Vilazodone, which was approved by the FDA in 2011, but it has been known to have circumvented the FDA requirement for two adequately conducted clinical trials showing a significant difference between drug and placebo since there is no limit to the number of trials, that can be conducted for that drug. There were a total of seven trials involving vilazodone, with the first five failing to show any significant benefit, while two managed to find a small but significant drug-placebo difference [31]. In contrast, the CANMAT guidelines for MDD recommends several new antidepressants including agomelatine and vortioxetine as first-line treatments due to their unique pharmacodynamic mechanisms and efficacy [31,32] and the popularity of these two antidepressants were on the rising trend.

### 4.3. Possible Explanations of Findings Related to Neurostimulation Therapies

The popularity of ECT has increased in the past 30 years with a positive index of change over 6 consecutive periods. ECT remains one of the most effective treatments for MDD, with response rates as high as 80%, and remission rates 50% or higher. It has the level 1 evidence (evidence from at least 1 RCT) in acute efficacy, maintenance efficacy, safety and tolerability [33]. The National Institute of Clinical Excellence (NICE) guidelines (UK) reported that the combination of ECT with pharmacotherapy was not shown to be superior to ECT alone [34]. For patients with treatment-resistant depression after the failure of two or more types of pharmacotherapy or psychotherapy, ECT is both clinical and cost-effective treatment option [35]. The efficacy of ECT has been correlated with increased network coherence in the default mode network, and depressed patients have been shown to have decreased network coherence [36]. ECT has other mechanisms of actions which antidepressants do not have. Patients with MDD have decreased cerebral blood flow in the frontal and limbic regions [37,38] and bilateral ECT caused hemodynamic changes in the bilateral prefrontal cortex [39]. Furthermore, ECT can acutely activate both the hypothalamic–pituitary–adrenal (HPA) axis and the dopamine system [40] and result in rapid treatment response.

The popularity of other neurostimulation therapies including rTMS, tDCS and DBS had increased, but these new therapies were not able to replace ECT. The NICE guidelines (UK) reported that ECT was found to be more clinically effective [41], and cost-effective than rTMS although rTMS has fewer side effects since it stimulates without inducing a seizure, and does not require anesthesia. rTMS has level 1 evidence (evidence from at least 1 RCT), in acute efficacy, safety and tolerability but level 3 evidence (opinions of respected authorities) in maintenance efficacy [33]. In a randomized sham-controlled rTMS study for treatment-resistant depression, the rTMS and sham groups attained similar remission rate [42]. tDCS applies a low-intensity, continuous current that alters cortical excitability but does not elicit an action potential [7]. tDCS has level 2 evidence (evidence from at least one well-designed cohort or case control study) in acute efficacy, safety, and tolerability but level 3 evidence (opinions of respected authorities) in maintenance efficacy [33]. VNS is a procedure that involves implantation of a device that stimulates the vagus nerve with electrical impulses. VNS has level two evidence in safety and tolerability and maintenance efficacy, but level 3 evidence (opinions of respected authorities) in acute efficacy [33]. The CANMAT guidelines classify tDCS and VNS as third-line treatment. DBS involves surgical implantation of a pulse generator in the brain, and it leads to adverse effects such as postoperative infection which is not encountered with other neurostimulation therapies [43]. As a result, the CANMAT guidelines classify DBS as an investigational treatment with level 3 evidence (opinions of respected authorities) in acute efficacy, safety and tolerability and maintenance efficacy [33]. Compared to ECT, the other neurostimulation treatments (e.g., tDCS, VNS, DBS) are not specifically used for the treatment of MDD, but these treatments have been used for treating other medical conditions including tDCS for headache, VNS for epilepsy and DBS for Parkinson’s disease. The recently developed neurostimulation modalities have not yet been able to replace ECT. 

There are several reasons to explain the decline in the publication of journal articles regarding the biological treatment of MDD from 2003 onwards. First, there have been significant advances in psychopharmacology which have offered better treatment options for those suffering from MDD. However, the field has now reached a steady state and improvements brought about by new antidepressants are expected to be less significant than those achieved 30 years ago [44]. Second, the global pharmaceutical industry has significantly decreased investment in new biological treatments for MDD [45], closed psychiatric laboratories, and decreased the size of research programs [46]. Due to lack of research funding for the development of new antidepressants, some researchers have proposed to use psychedelic properties of ketamine [47,48] and psilocybin [49] to treat MDD. Third, there are no validated biomarkers which can assess severity of depressive symptoms and judge the success of clinical trials objectively [46]. Researchers often measure the antidepressant effects by questionnaires [50]. As a result, there have been no significant breakthroughs in the research related to biological treatments for MDD.

### 4.4. Limitations

This study inherited the general limitations of the scientometric analysis. First, the scientometric indices could not differentiate between publications characterizing an antidepressant positively or negatively [11,22]. Furthermore, the number of publications might not reflect clinical practice and prescription pattern at a given time. As a result, the findings of this study should be interpreted in combination with meta-analyses. Based on the PI generated by scientometric analysis, fluoxetine was the subject of the highest number of publications. A recent network meta-analysis also suggested that fluoxetine is considered the best option among 14 antidepressants [51]. Second, the scientometric analysis is the study of the quantitative aspects of the process of science as a communication system [52] and it does not involve the assumption of a null hypothesis. As a result, the p-value is not applicable for scientometric analysis in this study. Finally, we conducted our search through the WOS only to avoid duplication of articles. However, as the number of articles was large (*n* = 355,116), there was a high possibility that these articles were indexed in other databases including PubMed, Embase, PsychInfo and Cochrane Library. Hence, articles included in this study are representative of the field.

## 5. Conclusions

This scientometric analysis represents the most comprehensive evidence on the popularity and change in prospects of 23 antidepressants and 5 neurostimulation therapies in the treatment of MDD from 1988 to 2017. Among 23 antidepressants, fluoxetine, paroxetine and sertraline were the subjects of the highest number of publications. The popularity of SSRI reached its peak in 1998–2002 while TCA and MAOI were replaced by other antidepressants and neurostimulation therapies. Among 5 neurostimulation therapies, the popularity of ECT had increased in the past 30 years with a positive index of change in six consecutive periods. Other antidepressants and neurostimulation therapies have not yet been able to replace ECT. 

## Figures and Tables

**Table 1:** Index of change and expectations of biological treatments for the major depressive disorder (MDD).

Name	Number of Article (Total)	PI ^a^	Index of Change (%) ^b^						Index of Expectations ^c^					
			1988–1992	1993–1997	1998–2002	2003–2007	2008–2012	2013–2017	1988–1992	1993–1997	1998–2002	2003–2007	2008–2012	2013–2017
fluoxetine	9244	2.60	-	**331.3**	**52.4**	6.6	9.9	1.3	-	3.60	4.83	4.55	4.47	3.97
paroxetine	4349	1.22	-	**322.8**	**100.9**	35.7	−17.0	−14.6	-	1.38	2.61	3.20	2.31	1.51
citalopram	3431	0.97	-	**597.0**	**125.2**	**55.6**	21.2	−13.1	-	0.86	1.60	2.09	2.15	1.74
sertraline	3349	0.94	-	**725.7**	**121.8**	21.8	1.0	3.0	-	1.09	1.96	2.15	1.43	1.31
amitriptyline	3177	0.89	-	101.0	−10.2	−13.7	−0.6	−0.4	-	1.69	1.14	1.07	0.76	0.70
venlafaxine	2849	0.80	-	**314.0**	**30.1**	−3.7	−24.7	−31.0	-	1.40	1.64	1.30	0.84	0.52
fluvoxamine	1944	0.55	-	**1733.3**	**240.0**	**82.6**	29.9	−11.0	-	0.47	1.24	1.95	2.09	1.58
clomipramine	1786	0.50	-	**130.7**	−5.1	−25.5	−14.4	−20.4	-	1.38	1.20	0.72	0.61	0.40
ECT	3554	1.00	-	76.2	**24.7**	20.3	29.3	19.0	-	1.98	2.47	2.97	3.84	4.57
rTMS	1371	0.39	-	-	**2014.3**	**62.8**	**68.0**	40.7	-	0.05	0.68	0.93	1.28	1.44
VNS	552	0.16	-	-	**580.0**	**250.0**	**55.5**	13.0	-	0.02	0.21	0.42	0.50	0.49
tDCS	438	0.12	-	-	-	**600.0**	**707.1**	**173.5**	-	-	0.01	0.09	0.44	0.92
DBS	984	0.28	-	-	**600.0**	**757.1**	**458.3**	**73.4**	-	0.01	0.05	0.24	0.88	1.43

Note: The index of change of a biological treatment is bolded if the index is higher than the index of change of all publications related to biological treatment of MDD within the same period, ^a^ Share % of all (355,116) field publications. ^b^ changes in the number of publications compared to the number of publications on a particular biological treatment in the previous 5 years. The index of change of a biological treatment is bolded if it is higher than the index of changes of all publications related to biological treatment of MDD during the same period. ^c^ Index of expectation assesses the ratio of the number of articles on a particular biological treatment to all articles related to the field of antidepressants or neurostimulation therapies in the top 20 journals covered by Web of Science over 5 years. Figures in bold indicate increases ratio higher than 1.0 in the field of antidepressants. The following antidepressants (not listed in the table) did not reach the threshold of 0.5% for the field of antidepressants: Escitalopram 0.45, Bupropion 0.46, Duloxetine 0.37, Mirtazapine 0.36, Nortriptyline 0.34, Trazodone 0.20, Reboxetine 0.17, moclobemide 0.17, nefazodone 0.14, Agomelatine 0.13, milnacipran 0.13, Vortioxetine 0.06, Desvenlafaxine 0.05, Vilazodone 0.03, Levomilnacipran 0.02.

**Table 2:** The PI for different classes of antidepressants and 5 neurostimulation therapies in different sub-specialties.

Classes of Antidepressants and Neurostimulation Therapies	PI in Psychiatry (%)	PI in Neurosciences & Neurology (%)	PI in Psychology (%)	PI in Pharmacology & Pharmacy (%)
SSRI	11.29	10.31	3.55	26.23
SNRI	1.36	1.01	0.43	3.33
TCA	0.91	0.75	0.31	1.88
ECT	2.55	1.40	0.46	1.09
rTMS	0.61	1.04	0.23	0.34
VNS	0.21	0.23	0.02	0.17
DBS	0.31	0.56	0.06	0.25
tDCS	0.14	0.29	0.03	0.11
**Total number of articles related to biological treatment of MDD**	**104,355**	**85,263**	**69,046**	**38,931**

Note: SSRI = Selective Serotonin Reuptake Inhibitors, SNRI = Serotonin Noradrenaline Reuptake Inhibitors, TCA = Tricyclic Antidepressants, ECT = Electroconvulsive therapy, rTMS = Repetitive Transcranial Magnetic Stimulation, VNS = Vagus Nerve Stimulation, DBS = Deep Brain Stimulation, tDCS = Transcranial Direct Current Stimulation, MDD = major depressive disorder.

**Table 3:** The PI of 23 antidepressants and neurostimulation therapies in Psychiatry (*n* = 104,335).

Biological Treatments for Major Depressive Disorder	Year of Approval	Number of Articles	PI %
**Antidepressants**			
fluoxetine	1987	4182	4.01
paroxetine	1996	2179	2.09
sertraline	1990	1736	1.66
citalopram	1998	1535	1.47
venlafaxine	1993	1423	1.36
amitriptyline	1961	1111	1.06
fluvoxamine	1994	1042	1.00
clomipramine	1970	946	0.91
escitalopram	2002	861	0.83
bupropion	1989	741	0.71
mirtazapine	1994	633	0.61
nortriptyline	1977	546	0.52
duloxetine	2004	485	0.46
moclobemide	2000	313	0.30
trazodone	1981	298	0.29
nefazodone	2003	285	0.27
reboxetine	1997	283	0.27
agomelatine	2009	219	0.21
milnacipran	1996	182	0.17
vortioxetine	2013	103	0.10
desvenlafaxine	2007	87	0.08
vilazodone	2011	45	0.04
levomilnacipran	2013	29	0.03
**Neurostimulation therapies**			
ECT	1954	2665	2.55
rTMS	1985	637	0.61
DBS	2009	327	0.31
VNS	2005	215	0.21
tDCS	2014	150	0.14

Note: the number of MDD article in Psychiatry *n* = 104,355.

**Table 4:** The index of change (IC) for journal articles on biological treatments of major depressive disorder in psychiatry and medical journals from 1988 to 2017.

Years	All Journal Articles Related to Biological Treatments for MDD		All Articles in Psychiatry Journals		All Articles in Medical Journals	
	Number	IC (%)	Number	IC (%)	Number	IC (%)
1988–1992	997	-	4679	-	1140814	-
1993–1997	2864	65.2	8751	46.5	1333570	14.5
1998–2002	4358	34.3	11593	24.5	1571304	15.1
2003–2007	5658	23.0	16485	29.7	1905855	17.6
2008–2012	7585	25.4	24137	31.7	2702338	29.5
2013–2017	8621	12.0	32096	24.8	3462317	22.0

**Table 5:** The index of change of popularity index for different classes of antidepressants and ECT.

Class of Antidepressants or Neurostimulation	The Index of Change of PI of Some Antidepressants and Neurostimulation Therapy (ECT)				
	(from 1988 to 2017) (%) *				
	1993–1997	1998–2002	2003–2007	2008–2012	2013–2017
TCA	25.6	−4.4	−51.6	−74.0	−85.0
MAOI	102.2	44.6	−38.8	−71.6	−81.3
ECT	−17.5	−15.4	−26.3	−37.9	−48.1
SSRI	77.1	142.6	116.2	50.0	−0.1
SNRI	93.9	107.3	44.6	−29.0	−65.5

* Reference period: 1998–1992.

## Supplementary Materials

Supplementary materials can be found at https://www.mdpi.com/1660-4601/16/13/2255/s1.
