# Supplementary material for: Indices of Change, Expectations, and Popularity of Biological Treatments for Major Depressive Disorder between 1988 and 2017: A Scientometric Analysis
Source: Int J Environ Res Public Health. 2019 Jun 26;16(13):2255. doi: 10.3390/ijerph16132255 (PMC6651662; doi:10.3390/ijerph16132255)
Supplement: Supplementary file 1 [file ijerph-16-02255-s001.zip › IJERPH Supplementary files/10. Supplementary 2.docx]

**The supplementary file 2. Number of publications of eight antidepressants with index of expectations > 1.0 and five neurostimulation therapies in the top 20 journals of biological treatments for major depressive disorder**

| **Fluoxetine** |  |  |
| --- | --- | --- |
| **Year** | **Journal** | **No of articles** |
| 2013-2017 | Journal Of Affective Disorders | 60 |
|  | Behavioural Brain Research | 49 |
|  | Pharmacology Biochemistry And Behavior | 47 |
|  | Psychopharmacology | 46 |
|  | Neuropharmacology | 40 |
|  | International Journal Of Neuropsychopharmacology | 35 |
|  | Pharmacological Reports | 28 |
|  | European Journal Of Pharmacology | 27 |
|  | European Neuropsychopharmacology | 27 |
|  | Neuroscience | 25 |
|  | Neuroscience Letters | 25 |
|  | Progress In Neuro Psychopharmacology Biological Psychiatry | 25 |
|  | Neuropsychopharmacology | 24 |
|  | Translational Psychiatry | 24 |
|  | Journal Of Clinical Psychopharmacology | 22 |
|  | Neuropsychiatric Disease And Treatment | 22 |
|  | Journal Of Clinical Psychiatry | 19 |
|  | Psychiatry Research | 17 |
|  | Behavioural Pharmacology | 16 |
|  | Frontiers In Cellular Neuroscience | 16 |
| 2008-2012 | Progress In Neuro Psychopharmacology Biological Psychiatry | 54 |
|  | Psychopharmacology | 52 |
|  | European Journal of Pharmacology | 45 |
|  | Pharmacology Biochemistry And Behavior | 44 |
|  | Journal of Affective Disorders | 43 |
|  | Journal of Clinical Psychiatry | 43 |
|  | Neuropsychopharmacology | 42 |
|  | International Journal of Neuropsychopharmacology | 39 |
|  | Neuropharmacology | 39 |
|  | Behavioural Brain Research | 36 |
|  | Neuroscience Letters | 31 |
|  | Journal of Psychopharmacology | 28 |
|  | Pharmacological Reports | 26 |
|  | Brain Research | 25 |
|  | European Neuropsychopharmacology | 22 |
|  | Biological Psychiatry | 21 |
|  | Journal of Clinical Psychopharmacology | 21 |
|  | International Clinical Psychopharmacology | 20 |
|  | Human Psychopharmacology Clinical And Experimental | 19 |
|  | Neuroscience | 18 |
| 2003-2007 | Journal Of Clinical Psychiatry | 93 |
|  | Psychopharmacology | 57 |
|  | Neuropsychopharmacology | 56 |
|  | Journal Of Clinical Psychopharmacology | 51 |
|  | Biological Psychiatry | 37 |
|  | Progress In Neuro Psychopharmacology Biological Psychiatry | 35 |
|  | European Journal Of Pharmacology | 34 |
|  | Journal Of Affective Disorders | 31 |
|  | Journal Of Child And Adolescent Psychopharmacology | 31 |
|  | International Clinical Psychopharmacology | 30 |
|  | Journal Of The American Academy Of Child And Adolescent Psychiatry | 30 |
|  | Pharmacology Biochemistry And Behavior | 30 |
|  | Neuropharmacology | 24 |
|  | Brain Research | 22 |
|  | International Journal Of Neuropsychopharmacology | 22 |
|  | Human Psychopharmacology Clinical And Experimental | 21 |
|  | Depression And Anxiety | 20 |
|  | American Journal Of Psychiatry | 19 |
|  | European Neuropsychopharmacology | 19 |
|  | Journal Of Psychopharmacology | 18 |
| 1998-2002 | Journal Of Clinical Psychiatry | 75 |
|  | Journal Of Clinical Psychopharmacology | 63 |
|  | Psychopharmacology | 61 |
|  | Journal Of Affective Disorders | 50 |
|  | International Clinical Psychopharmacology | 46 |
|  | Neuropsychopharmacology | 40 |
|  | American Journal Of Psychiatry | 37 |
|  | Biological Psychiatry | 37 |
|  | CNS Drugs | 31 |
|  | European Journal Of Pharmacology | 31 |
|  | Journal Of Psychopharmacology | 30 |
|  | Annals Of Pharmacotherapy | 27 |
|  | Pharmacology Biochemistry And Behavior | 27 |
|  | Journal Of Pharmacology And Experimental Therapeutics | 26 |
|  | Neuropharmacology | 26 |
|  | European Neuropsychopharmacology | 25 |
|  | Human Psychopharmacology Clinical And Experimental | 24 |
|  | Brain Research | 20 |
|  | British Journal Of Pharmacology | 20 |
|  | British Journal Of Psychiatry | 20 |
| 1993-1997 | Journal Of Clinical Psychiatry | 68 |
|  | Journal Of Clinical Psychopharmacology | 60 |
|  | International Clinical Psychopharmacology | 57 |
|  | Journal Of Affective Disorders | 33 |
|  | Human Psychopharmacology Clinical And Experimental | 32 |
|  | Cns Drugs | 31 |
|  | Psychopharmacology | 28 |
|  | European Journal Of Pharmacology | 22 |
|  | Acta Psychiatrica Scandinavica | 19 |
|  | Journal Of Pharmacology And Experimental Therapeutics | 19 |
|  | Journal Of Psychopharmacology | 19 |
|  | Neuropsychopharmacology | 19 |
|  | American Journal Of Psychiatry | 18 |
|  | Pharmacopsychiatry | 18 |
|  | Biological Psychiatry | 17 |
|  | Progress In Neuro Psychopharmacology Biological Psychiatry | 16 |
|  | Psychiatric Annals | 15 |
|  | Psychopharmacology Bulletin | 15 |
|  | Psychiatric Clinics Of North America | 14 |
|  | Annals Of Pharmacotherapy | 12 |
| 1988-1992 | Journal Of Clinical Psychiatry | 35 |
|  | International Clinical Psychopharmacology | 28 |
|  | British Journal Of Psychiatry | 8 |
|  | Journal Of Clinical Psychopharmacology | 8 |
|  | Psychopharmacology | 8 |
|  | Journal Of Affective Disorders | 7 |
|  | Psychopharmacology Bulletin | 6 |
|  | American Journal Of Psychiatry | 5 |
|  | Biological Psychiatry | 5 |
|  | Human Psychopharmacology Clinical And Experimental | 5 |
|  | Journal Of Pharmacology And Experimental Therapeutics | 5 |
|  | Clinical Pharmacology Therapeutics | 4 |
|  | Current Therapeutic Research Clinical And Experimental | 4 |
|  | Drugs | 4 |
|  | Journal Of The American Academy Of Child And Adolescent Psychiatry | 4 |
|  | Psychiatric Clinics Of North America | 4 |
|  | ACTA Psychiatrica Scandinavica | 3 |
|  | Archives Of General Psychiatry | 3 |
|  | Brain Research | 3 |
|  | Canadian Journal Of Psychiatry Revue Canadienne De Psychiatrie | 3 |

| **Paroxetine** |  |  |
| --- | --- | --- |
| **Year** | **Journal** | **No of articles** |
| 2013-2017 | Neuropsychiatric Disease And Treatment | 26 |
|  | Journal Of Clinical Psychiatry | 16 |
|  | Journal Of Affective Disorders | 15 |
|  | Journal Of Clinical Psychopharmacology | 15 |
|  | Pharmacology Biochemistry And Behavior | 14 |
|  | Cochrane Database Of Systematic Reviews | 13 |
|  | Human Psychopharmacology Clinical And Experimental | 12 |
|  | International Journal Of Neuropsychopharmacology | 12 |
|  | Clinical Neuropharmacology | 11 |
|  | Psychopharmacology | 11 |
|  | Psychiatry Research | 10 |
|  | European Journal Of Pharmacology | 9 |
|  | Neuropharmacology | 9 |
|  | American Journal Of Psychiatry | 8 |
|  | Annals Of Pharmacotherapy | 8 |
|  | International Clinical Psychopharmacology | 8 |
|  | Journal Of Psychopharmacology | 8 |
|  | Bmc Psychiatry | 7 |
|  | Cns Drugs | 7 |
|  | Depression And Anxiety | 7 |
| 2008-2012 | Journal Of Clinical Psychiatry | 33 |
|  | Journal Of Affective Disorders | 31 |
|  | Journal Of Clinical Psychopharmacology | 30 |
|  | Progress In Neuro Psychopharmacology Biological Psychiatry | 24 |
|  | Journal Of Psychopharmacology | 22 |
|  | Psychopharmacology | 19 |
|  | Human Psychopharmacology Clinical And Experimental | 18 |
|  | Journal Of Sexual Medicine | 17 |
|  | Neuropsychopharmacology | 17 |
|  | European Journal Of Pharmacology | 16 |
|  | Neuroscience Letters | 16 |
|  | Journal Of Psychiatric Research | 14 |
|  | European Neuropsychopharmacology | 13 |
|  | International Clinical Psychopharmacology | 13 |
|  | International Journal Of Neuropsychopharmacology | 12 |
|  | Psychiatry Research Neuroimaging | 11 |
|  | Cochrane Database Of Systematic Reviews | 10 |
|  | Psychiatry And Clinical Neurosciences | 10 |
|  | Drug Safety | 9 |
|  | Psychiatry Research | 8 |
| 2003-2007 | Journal Of Clinical Psychiatry | 72 |
|  | Psychopharmacology | 35 |
|  | Journal Of Clinical Psychopharmacology | 33 |
|  | Progress In Neuro Psychopharmacology Biological Psychiatry | 31 |
|  | International Clinical Psychopharmacology | 28 |
|  | Biological Psychiatry | 27 |
|  | Journal Of Affective Disorders | 24 |
|  | Neuropsychopharmacology | 23 |
|  | International Journal Of Neuropsychopharmacology | 21 |
|  | European Neuropsychopharmacology | 20 |
|  | Journal Of Child And Adolescent Psychopharmacology | 20 |
|  | Annals Of Pharmacotherapy | 19 |
|  | Depression And Anxiety | 19 |
|  | Journal Of Psychopharmacology | 17 |
|  | Human Psychopharmacology Clinical And Experimental | 16 |
|  | Pharmacopsychiatry | 16 |
|  | Journal Of Psychiatric Research | 14 |
|  | International Journal Of Psychiatry In Clinical Practice | 13 |
|  | Psychiatry Research | 13 |
|  | Archives Of General Psychiatry | 12 |
| 1998-2002 | Psychiatry | 475 |
|  | Pharmacology Pharmacy | 403 |
|  | Neurosciences Neurology | 312 |
|  | Psychology | 103 |
|  | General Internal Medicine | 35 |
|  | Biochemistry Molecular Biology | 30 |
|  | Toxicology | 30 |
|  | Geriatrics Gerontology | 20 |
|  | Pediatrics | 19 |
|  | Behavioral Sciences | 16 |
|  | Chemistry | 16 |
|  | Public Environmental Occupational Health | 16 |
|  | Research Experimental Medicine | 14 |
|  | Health Care Sciences Services | 13 |
|  | Radiology Nuclear Medicine Medical Imaging | 9 |
|  | Endocrinology Metabolism | 8 |
|  | Medical Laboratory Technology | 7 |
|  | Cardiovascular System Cardiology | 6 |
|  | Cell Biology | 5 |
|  | Oncology | 5 |
| 1993-1997 | Psychopharmacology | 21 |
|  | Journal Of Clinical Psychopharmacology | 20 |
|  | Human Psychopharmacology Clinical And Experimental | 17 |
|  | Journal Of Affective Disorders | 12 |
|  | Journal Of Clinical Psychiatry | 12 |
|  | Pharmacopsychiatry | 12 |
|  | Acta Psychiatrica Scandinavica | 11 |
|  | Biological Psychiatry | 11 |
|  | International Clinical Psychopharmacology | 11 |
|  | Brain Research | 8 |
|  | British Journal Of Pharmacology | 8 |
|  | Journal Of Psychopharmacology | 8 |
|  | Neuropsychopharmacology | 8 |
|  | American Journal Of Medicine | 7 |
|  | Journal Of Pharmacology And Experimental Therapeutics | 7 |
|  | Psychiatry Research | 7 |
|  | Annals Of Pharmacotherapy | 6 |
|  | Clinical Pharmacokinetics | 6 |
|  | European Neuropsychopharmacology | 6 |
|  | Naunyn Schmiedebergs Archives Of Pharmacology | 6 |

| **Citalopram** |  | | |  |  |
| --- | --- | --- | --- | --- | --- |
| **Year** | **Journal** | | | **No of articles** |  |
| 2013-2017 | Journal Of Affective Disorders | | | 26 |  |
|  | Psychopharmacology | | | 25 |  |
|  | European Neuropsychopharmacology | | | 24 |  |
|  | Journal Of Clinical Psychiatry | | | 15 |  |
|  | Neuropsychopharmacology | | | 15 |  |
|  | International Journal Of Neuropsychopharmacology | | | 14 |  |
|  | International Clinical Psychopharmacology | | | 13 |  |
|  | Neuropharmacology | | | 13 |  |
|  | Journal Of Clinical Psychopharmacology | | | 12 |  |
|  | Neuropsychiatric Disease And Treatment | | | 12 |  |
|  | Progress In Neuro Psychopharmacology Biological Psychiatry | | | 11 |  |
|  | Cochrane Database Of Systematic Reviews | | | 10 |  |
|  | European Journal Of Pharmacology | | | 10 |  |
|  | Pharmacological Reports | | | 10 |  |
|  | Journal Of Neural Transmission | | | 9 |  |
|  | Journal Of Psychiatric Research | | | 9 |  |
|  | Psychological Medicine | | | 9 |  |
|  | American Journal Of Psychiatry | | | 8 |  |
|  | Journal Of Psychopharmacology | | | 8 |  |
|  | Neuroscience | | | 8 |  |
| 2008-2012 | Psychopharmacology | | | 36 |  |
|  | International Journal Of Neuropsychopharmacology | | | 23 |  |
|  | Journal Of Clinical Psychiatry | | | 21 |  |
|  | Journal Of Psychopharmacology | | | 21 |  |
|  | Progress In Neuro Psychopharmacology Biological Psychiatry | | | 21 |  |
|  | Journal Of Clinical Psychopharmacology | | | 19 |  |
|  | Neuropsychopharmacology | | | 18 |  |
|  | European Neuropsychopharmacology | | | 16 |  |
|  | Journal Of Affective Disorders | | | 16 |  |
|  | European Journal Of Pharmacology | | | 15 |  |
|  | Neuroscience Letters | | | 14 |  |
|  | Psychiatry Research | | | 14 |  |
|  | Pharmacology Biochemistry And Behavior | | | 13 |  |
|  | Biological Psychiatry | | | 12 |  |
|  | International Clinical Psychopharmacology | | | 12 |  |
|  | Journal Of Psychiatric Research | | | 11 |  |
|  | Human Psychopharmacology Clinical And Experimental | | | 10 |  |
|  | Neurochemistry International | | | 10 |  |
|  | Behavioural Brain Research | | | 9 |  |
|  | Cochrane Database Of Systematic Reviews | | | 9 |  |
| 2003-2007 | Journal Of Clinical Psychiatry | | | 36 |  |
|  | Psychopharmacology | | | 35 |  |
|  | Neuropsychopharmacology | | | 27 |  |
|  | European Journal Of Pharmacology | | | 18 |  |
|  | Progress In Neuro Psychopharmacology Biological Psychiatry | | | 18 |  |
|  | American Journal Of Psychiatry | | | 17 |  |
|  | Journal Of Clinical Psychopharmacology | | | 17 |  |
|  | Journal Of Affective Disorders | | | 15 |  |
|  | Pharmacopsychiatry | | | 14 |  |
|  | Biological Psychiatry | | | 13 |  |
|  | European Neuropsychopharmacology | | | 12 |  |
|  | International Clinical Psychopharmacology | | | 12 |  |
|  | International Journal Of Neuropsychopharmacology | | | 12 |  |
|  | Annals Of Pharmacotherapy | | | 10 |  |
|  | Journal Of Psychopharmacology | | | 10 |  |
|  | Neuropsychobiology | | | 10 |  |
|  | Behavioural Pharmacology | | | 9 |  |
|  | Cns Drugs | | | 9 |  |
|  | Depression And Anxiety | | | 9 |  |
|  | Human Psychopharmacology Clinical And Experimental | | | 9 |  |
| 1998-2002 | Psychopharmacology | | | 28 |  |
|  | European Journal Of Pharmacology | | | 20 |  |
|  | International Clinical Psychopharmacology | | | 19 |  |
|  | Journal Of Clinical Psychopharmacology | | | 17 |  |
|  | Journal Of Clinical Psychiatry | | | 14 |  |
|  | Human Psychopharmacology Clinical And Experimental | | | 12 |  |
|  | British Journal Of Pharmacology | | | 11 |  |
|  | Pharmacopsychiatry | | | 11 |  |
|  | European Neuropsychopharmacology | | | 10 |  |
|  | Progress In Neuro Psychopharmacology Biological Psychiatry | | | 10 |  |
|  | Cns Drugs | | | 9 |  |
|  | Journal Of Pharmacology And Experimental Therapeutics | | | 9 |  |
|  | Journal Of Psychopharmacology | | | 9 |  |
|  | Neuropharmacology | | | 9 |  |
|  | Synapse | | | 9 |  |
|  | Biological Psychiatry | | | 8 |  |
|  | Clinical Neuropharmacology | | | 8 |  |
|  | Journal Of Neurochemistry | | | 8 |  |
|  | Naunyn Schmiedebergs Archives Of Pharmacology | | | 8 |  |
|  | Therapeutic Drug Monitoring | | | 7 |  |
| 1993-1997 | European Journal Of Pharmacology | | | 15 |  |
|  | Psychopharmacology | | | 13 |  |
|  | International Clinical Psychopharmacology | | | 12 |  |
|  | Neuropharmacology | | | 9 |  |
|  | Acta Psychiatrica Scandinavica | | | 8 |  |
|  | Journal Of Neurochemistry | | | 8 |  |
|  | Naunyn Schmiedebergs Archives Of Pharmacology | | | 7 |  |
|  | Therapeutic Drug Monitoring | | | 6 |  |
|  | Clinical Pharmacokinetics | | | 5 |  |
|  | Cns Drugs | | | 5 |  |
|  | Journal Of Pharmacology And Experimental Therapeutics | | | 5 |  |
|  | Neuropsychopharmacology | | | 5 |  |
|  | Human Psychopharmacology Clinical And Experimental | | | 4 |  |
|  | Journal Of Neural Transmission | | | 4 |  |
|  | Pharmacology Biochemistry And Behavior | | | 4 |  |
|  | Pharmacopsychiatry | | | 4 |  |
|  | Clinical Neuropharmacology | | | 3 |  |
|  | European Neuropsychopharmacology | | | 3 |  |
|  | Homeostasis In Health And Disease | | | 3 |  |
|  | Journal Of Clinical Psychopharmacology | | | 3 |  |
| 1988-1992 | Journal Of Neural Transmission General Section | | | 3 |  |
|  | Neuropharmacology | | | 3 |  |
|  | Neuropsychopharmacology | | | 3 |  |
|  | Brain Research | | | 2 |  |
|  | Clinical Pharmacology Therapeutics | | | 2 |  |
|  | European Journal Of Pharmacology | | | 2 |  |
|  | Acta Psychiatrica Scandinavica | | | 1 |  |
|  | Biochemical Pharmacology | | | 1 |  |
|  | British Journal Of Clinical Pharmacology | | | 1 |  |
|  | British Journal Of Pharmacology | | | 1 |  |
|  | Drugs | | | 1 |  |
|  | Fundamental Clinical Pharmacology | | | 1 |  |
|  | International Clinical Psychopharmacology | | | 1 |  |
|  | Journal Of Cardiovascular Pharmacology | | | 1 |  |
|  | Journal Of Neurochemistry | | | 1 |  |
|  | Journal Of Neurophysiology | | | 1 |  |
|  | Journal Of Pharmacology And Experimental Therapeutics | | | 1 |  |
|  | Journal Of Pharmacy And Pharmacology | | | 1 |  |
|  | Neuroendocrinology | | | 1 |  |
|  | Pharmacology Toxicology | | | 1 |  |
| **Sertaline** | |  |  | | |
| **Year** | | **Journal** | **No of articles** | | |
| 2013-2017 | | Journal Of Affective Disorders | 25 | | |
|  | | Journal Of Clinical Psychiatry | 16 | | |
|  | | Cochrane Database Of Systematic Reviews | 12 | | |
|  | | Journal Of Clinical Psychopharmacology | 11 | | |
|  | | Neuropsychiatric Disease And Treatment | 11 | | |
|  | | American Journal Of Geriatric Psychiatry | 10 | | |
|  | | International Journal Of Neuropsychopharmacology | 10 | | |
|  | | Psychopharmacology | 10 | | |
|  | | Bmc Psychiatry | 9 | | |
|  | | European Neuropsychopharmacology | 9 | | |
|  | | Human Psychopharmacology Clinical And Experimental | 9 | | |
|  | | British Journal Of Psychiatry | 8 | | |
|  | | Depression And Anxiety | 8 | | |
|  | | International Clinical Psychopharmacology | 8 | | |
|  | | Journal Of Pharmaceutical And Biomedical Analysis | 8 | | |
|  | | Annals Of Pharmacotherapy | 7 | | |
|  | | Psychiatry Research | 7 | | |
|  | | American Journal Of Psychiatry | 6 | | |
|  | | Jama Psychiatry | 6 | | |
|  | | Psychiatry Investigation | 6 | | |
| 2008-2012 | | Journal Of Clinical Psychiatry | 26 | | |
|  | | Journal Of Affective Disorders | 19 | | |
|  | | Journal Of Clinical Psychopharmacology | 19 | | |
|  | | Progress In Neuro Psychopharmacology Biological Psychiatry | 18 | | |
|  | | Journal Of Psychopharmacology | 17 | | |
|  | | International Clinical Psychopharmacology | 12 | | |
|  | | Archives Of General Psychiatry | 10 | | |
|  | | Human Psychopharmacology Clinical And Experimental | 9 | | |
|  | | Journal Of Psychiatric Research | 8 | | |
|  | | Psychiatry Research | 8 | | |
|  | | Cochrane Database Of Systematic Reviews | 7 | | |
|  | | Depression And Anxiety | 7 | | |
|  | | European Journal Of Pharmacology | 7 | | |
|  | | Journal Of Chromatography B Analytical Technologies In The Biomedical And Life Sciences | 7 | | |
|  | | Neuropsychiatric Disease And Treatment | 7 | | |
|  | | Pharmacoepidemiology And Drug Safety | 7 | | |
|  | | Pharmacotherapy | 7 | | |
|  | | World Journal Of Biological Psychiatry | 7 | | |
|  | | American Journal Of Geriatric Psychiatry | 6 | | |
|  | | American Journal Of Psychiatry | 6 | | |
| 2003-2007 | | Journal Of Clinical Psychiatry | 68 | | |
|  | | Journal Of Clinical Psychopharmacology | 39 | | |
|  | | International Clinical Psychopharmacology | 22 | | |
|  | | Progress In Neuro Psychopharmacology Biological Psychiatry | 17 | | |
|  | | Journal Of Child And Adolescent Psychopharmacology | 16 | | |
|  | | Psychopharmacology | 15 | | |
|  | | Journal Of Affective Disorders | 13 | | |
|  | | Cns Spectrums | 12 | | |
|  | | Human Psychopharmacology Clinical And Experimental | 12 | | |
|  | | Journal Of The American Academy Of Child And Adolescent Psychiatry | 12 | | |
|  | | Depression And Anxiety | 11 | | |
|  | | International Journal Of Neuropsychopharmacology | 11 | | |
|  | | Psychosomatic Medicine | 11 | | |
|  | | American Journal Of Geriatric Psychiatry | 10 | | |
|  | | Biological Psychiatry | 10 | | |
|  | | Acta Psychiatrica Scandinavica | 9 | | |
|  | | American Journal Of Psychiatry | 9 | | |
|  | | Cns Drugs | 9 | | |
|  | | Archives Of General Psychiatry | 8 | | |
|  | | European Neuropsychopharmacology | 8 | | |
| 1998-2002 | | Journal Of Clinical Psychiatry | 38 | | |
|  | | Journal Of Clinical Psychopharmacology | 26 | | |
|  | | International Clinical Psychopharmacology | 24 | | |
|  | | Journal Of Affective Disorders | 24 | | |
|  | | Journal Of Psychopharmacology | 18 | | |
|  | | Psychopharmacology | 17 | | |
|  | | Annals Of Pharmacotherapy | 16 | | |
|  | | Biological Psychiatry | 14 | | |
|  | | Human Psychopharmacology Clinical And Experimental | 13 | | |
|  | | Cns Drugs | 12 | | |
|  | | Clinical Therapeutics | 11 | | |
|  | | American Journal Of Psychiatry | 10 | | |
|  | | Journal Of Child And Adolescent Psychopharmacology | 10 | | |
|  | | Depression And Anxiety | 9 | | |
|  | | Journal Of The American Academy Of Child And Adolescent Psychiatry | 9 | | |
|  | | Pharmacopsychiatry | 9 | | |
|  | | American Journal Of Geriatric Psychiatry | 8 | | |
|  | | British Journal Of Psychiatry | 8 | | |
|  | | Jama Journal Of The American Medical Association | 8 | | |
|  | | Current Opinion In Psychiatry | 7 | | |
| 1993-1997 | | Journal Of Clinical Psychiatry | 31 | | |
|  | | Journal Of Clinical Psychopharmacology | 14 | | |
|  | | Human Psychopharmacology Clinical And Experimental | 13 | | |
|  | | International Clinical Psychopharmacology | 12 | | |
|  | | British Journal Of Clinical Pharmacology | 10 | | |
|  | | Journal Of Psychopharmacology | 10 | | |
|  | | Journal Of Child And Adolescent Psychopharmacology | 8 | | |
|  | | Clinical Pharmacokinetics | 7 | | |
|  | | Psychopharmacology | 7 | | |
|  | | Journal Of Affective Disorders | 6 | | |
|  | | American Journal Of Medicine | 5 | | |
|  | | American Journal Of Psychiatry | 5 | | |
|  | | Pharmacotherapy | 5 | | |
|  | | Psychopharmacology Bulletin | 5 | | |
|  | | Acta Psychiatrica Scandinavica | 4 | | |
|  | | British Journal Of Psychiatry | 4 | | |
|  | | Clinical Therapeutics | 4 | | |
|  | | Cns Drugs | 4 | | |
|  | | European Psychiatry | 4 | | |
|  | | Journal Of Pharmacology And Experimental Therapeutics | 4 | | |
| 1988-1992 | | International Clinical Psychopharmacology | 6 | | |
|  | | Journal Of Clinical Psychiatry | 5 | | |
|  | | British Journal Of Pharmacology | 2 | | |
|  | | British Journal Of Psychiatry | 2 | | |
|  | | Journal Of Clinical Psychopharmacology | 2 | | |
|  | | Neuropsychopharmacology | 2 | | |
|  | | Psychopharmacology | 2 | | |
|  | | Clinical Pharmacy | 1 | | |
|  | | Dicp The Annals Of Pharmacotherapy | 1 | | |
|  | | Drug Development Research | 1 | | |
|  | | Drug Investigation | 1 | | |
|  | | Drugs | 1 | | |
|  | | European Journal Of Pharmacology | 1 | | |
|  | | Hospital And Community Psychiatry | 1 | | |
|  | | Hospital Formulary | 1 | | |
|  | | Human Psychopharmacology Clinical And Experimental | 1 | | |
|  | | Journal Of Neural Transmission General Section | 1 | | |
|  | | Journal Of Pharmacology And Experimental Therapeutics | 1 | | |
|  | | Pharmacology Biochemistry And Behavior | 1 | | |
|  | | Progress In Brain Research | 1 | | |

| **Amitriptyline** |  | |  | |
| --- | --- | --- | --- | --- |
| **Year** | **Journal** | **No of articles** | |  |
| 2013-2017 | Cochrane Database Of Systematic Reviews | | 18 | |
|  | Journal Of Chromatography A | | 8 | |
|  | Pharmacological Reports | | 7 | |
|  | European Journal Of Pharmacology | | 6 | |
|  | Progress In Neuro Psychopharmacology Biological Psychiatry | | 6 | |
|  | Current Pain And Headache Reports | | 5 | |
|  | Psychopharmacology | | 5 | |
|  | Behavioural Brain Research | | 4 | |
|  | Biological Pharmaceutical Bulletin | | 4 | |
|  | Clinical Neuropharmacology | | 4 | |
|  | Farmacia | | 4 | |
|  | Human Experimental Toxicology | | 4 | |
|  | Journal Of Affective Disorders | | 4 | |
|  | Journal Of Clinical Psychopharmacology | | 4 | |
|  | Pain | | 4 | |
|  | Pain Medicine | | 4 | |
|  | Pain Practice | | 4 | |
|  | Basic Clinical Pharmacology Toxicology | | 3 | |
|  | Bmj British Medical Journal | | 3 | |
|  | Brazilian Journal Of Pharmaceutical Sciences | | 3 | |
| 2008-2012 | Cochrane Database Of Systematic Reviews | | 11 | |
|  | Behavioural Brain Research | | 8 | |
|  | Journal Of Affective Disorders | | 8 | |
|  | Anesthesia And Analgesia | | 7 | |
|  | Clinical Toxicology | | 7 | |
|  | European Journal Of Pharmacology | | 7 | |
|  | Pharmacopsychiatry | | 7 | |
|  | Brain Research | | 6 | |
|  | Human Experimental Toxicology | | 6 | |
|  | Cns Drugs | | 5 | |
|  | Journal Of Clinical Psychiatry | | 5 | |
|  | British Journal Of Clinical Pharmacology | | 4 | |
|  | British Journal Of Pharmacology | | 4 | |
|  | Chromatographia | | 4 | |
|  | Current Treatment Options In Neurology | | 4 | |
|  | Human Psychopharmacology Clinical And Experimental | | 4 | |
|  | Journal Of Chromatography A | | 4 | |
|  | Journal Of Pharmaceutical Sciences | | 4 | |
|  | Journal Of Pharmacological Sciences | | 4 | |
|  | Journal Of Psychiatric Research | | 4 | |
| 2003-2007 | Pharmacopsychiatry | | 18 | |
|  | Journal Of Clinical Psychopharmacology | | 12 | |
|  | Anesthesia And Analgesia | | 10 | |
|  | European Journal Of Pharmacology | | 10 | |
|  | Journal Of Clinical Psychiatry | | 10 | |
|  | Pain | | 10 | |
|  | Psychopharmacology | | 10 | |
|  | Human Psychopharmacology Clinical And Experimental | | 8 | |
|  | Neuropsychopharmacology | | 8 | |
|  | Anesthesiology | | 6 | |
|  | Cochrane Database Of Systematic Reviews | | 6 | |
|  | European Neuropsychopharmacology | | 6 | |
|  | International Journal Of Neuropsychopharmacology | | 6 | |
|  | Journal Of Pain | | 6 | |
|  | Journal Of Pharmacology And Experimental Therapeutics | | 6 | |
|  | Neuropharmacology | | 6 | |
|  | Neuroscience Letters | | 6 | |
|  | Biological Psychiatry | | 5 | |
|  | Cephalalgia | | 5 | |
|  | Current Pharmaceutical Design | | 5 | |
| 1998-2002 | Journal Of Affective Disorders | | 18 | |
|  | Psychopharmacology | | 13 | |
|  | British Journal Of Pharmacology | | 10 | |
|  | Journal Of Clinical Psychopharmacology | | 10 | |
|  | Pharmacopsychiatry | | 10 | |
|  | International Clinical Psychopharmacology | | 9 | |
|  | Pain | | 9 | |
|  | European Neuropsychopharmacology | | 8 | |
|  | Human Psychopharmacology Clinical And Experimental | | 8 | |
|  | Neuropsychopharmacology | | 8 | |
|  | Pharmacology Biochemistry And Behavior | | 8 | |
|  | Clinical Therapeutics | | 7 | |
|  | Cns Drugs | | 7 | |
|  | European Journal Of Pharmacology | | 7 | |
|  | Journal Of Clinical Psychiatry | | 7 | |
|  | Electrophoresis | | 6 | |
|  | Headache | | 6 | |
|  | Journal Of Pharmacy And Pharmacology | | 6 | |
|  | Journal Of Psychopharmacology | | 6 | |
|  | Therapeutic Drug Monitoring | | 6 | |
| 1993-1997 | International Clinical Psychopharmacology | | 30 | |
|  | Human Psychopharmacology Clinical And Experimental | | 21 | |
|  | Pharmacopsychiatry | | 19 | |
|  | Acta Psychiatrica Scandinavica | | 18 | |
|  | Journal Of Affective Disorders | | 14 | |
|  | Journal Of Clinical Psychopharmacology | | 14 | |
|  | Biological Psychiatry | | 12 | |
|  | Psychopharmacology | | 12 | |
|  | Progress In Neuro Psychopharmacology Biological Psychiatry | | 11 | |
|  | Cns Drugs | | 10 | |
|  | European Journal Of Pharmacology | | 10 | |
|  | Journal Of Clinical Psychiatry | | 10 | |
|  | Pharmacology Biochemistry And Behavior | | 10 | |
|  | European Psychiatry | | 9 | |
|  | Current Therapeutic Research Clinical And Experimental | | 8 | |
|  | Journal Of Psychopharmacology | | 8 | |
|  | Neuropsychobiology | | 8 | |
|  | Pain | | 8 | |
|  | British Journal Of Pharmacology | | 7 | |
|  | Psychiatry Research | | 7 | |
| 1988-1992 | Journal Of Affective Disorders | | 13 | |
|  | International Clinical Psychopharmacology | | 12 | |
|  | Psychopharmacology | | 11 | |
|  | Acta Psychiatrica Scandinavica | | 9 | |
|  | Journal Of Clinical Psychiatry | | 9 | |
|  | Journal Of Clinical Psychopharmacology | | 8 | |
|  | Pain | | 7 | |
|  | Therapeutic Drug Monitoring | | 7 | |
|  | British Journal Of Psychiatry | | 6 | |
|  | Drugs | | 6 | |
|  | Human Psychopharmacology Clinical And Experimental | | 6 | |
|  | Current Therapeutic Research Clinical And Experimental | | 5 | |
|  | European Journal Of Clinical Pharmacology | | 5 | |
|  | Journal Of Neurochemistry | | 5 | |
|  | Neuropsychobiology | | 5 | |
|  | Pharmacopsychiatry | | 5 | |
|  | Progress In Neuro Psychopharmacology Biological Psychiatry | | 5 | |
|  | European Journal Of Pharmacology | | 4 | |
|  | Neuropsychopharmacology | | 4 | |
|  | American Journal Of Psychiatry | | 3 | |

| **Venlafaxine** |  |  |
| --- | --- | --- |
| **Year** | **Journal** | **No of articles** |
| 2013-2017 | Journal Of Affective Disorders | 20 |
|  | International Clinical Psychopharmacology | 17 |
|  | Pharmacopsychiatry | 17 |
|  | Journal Of Clinical Psychopharmacology | 15 |
|  | International Journal Of Neuropsychopharmacology | 14 |
|  | Journal Of Clinical Psychiatry | 14 |
|  | Neuropsychiatric Disease And Treatment | 14 |
|  | European Neuropsychopharmacology | 13 |
|  | Human Psychopharmacology Clinical And Experimental | 13 |
|  | Psychopharmacology | 13 |
|  | Journal Of Psychiatric Research | 12 |
|  | Cochrane Database Of Systematic Reviews | 11 |
|  | European Journal Of Pharmacology | 10 |
|  | Pharmacological Reports | 9 |
|  | American Journal Of Psychiatry | 8 |
|  | Journal Of Psychopharmacology | 8 |
|  | Annals Of Pharmacotherapy | 7 |
|  | British Journal Of Psychiatry | 7 |
|  | Clinical Neuropharmacology | 7 |
|  | Progress In Neuro Psychopharmacology Biological Psychiatry | 7 |
| 2008-2012 | Journal Of Clinical Psychiatry | 32 |
|  | Journal Of Clinical Psychopharmacology | 25 |
|  | International Clinical Psychopharmacology | 24 |
|  | Journal Of Psychopharmacology | 23 |
|  | Cns Spectrums | 20 |
|  | International Journal Of Neuropsychopharmacology | 19 |
|  | Journal Of Affective Disorders | 17 |
|  | Cns Drugs | 15 |
|  | European Neuropsychopharmacology | 15 |
|  | Human Psychopharmacology Clinical And Experimental | 13 |
|  | Journal Of Psychiatric Research | 13 |
|  | Current Medical Research And Opinion | 12 |
|  | Expert Opinion On Pharmacotherapy | 12 |
|  | Annals Of Pharmacotherapy | 11 |
|  | Clinical Therapeutics | 11 |
|  | Progress In Neuro Psychopharmacology Biological Psychiatry | 11 |
|  | Psychopharmacology | 11 |
|  | Acta Psychiatrica Scandinavica | 10 |
|  | Neuropsychiatric Disease And Treatment | 10 |
|  | American Journal Of Psychiatry | 8 |
| 2003-2007 | Journal Of Clinical Psychiatry | 50 |
|  | Journal Of Clinical Psychopharmacology | 26 |
|  | International Clinical Psychopharmacology | 22 |
|  | Progress In Neuro Psychopharmacology Biological Psychiatry | 18 |
|  | Human Psychopharmacology Clinical And Experimental | 16 |
|  | Psychopharmacology | 16 |
|  | Biological Psychiatry | 13 |
|  | Annals Of Pharmacotherapy | 12 |
|  | International Journal Of Neuropsychopharmacology | 12 |
|  | Journal Of Psychopharmacology | 12 |
|  | Cns Drugs | 11 |
|  | European Journal Of Pharmacology | 11 |
|  | International Journal Of Psychiatry In Clinical Practice | 11 |
|  | Depression And Anxiety | 10 |
|  | Expert Opinion On Pharmacotherapy | 10 |
|  | Journal Of Affective Disorders | 10 |
|  | Current Medical Research And Opinion | 9 |
|  | American Journal Of Psychiatry | 8 |
|  | Journal Of Pharmacology And Experimental Therapeutics | 8 |
|  | British Journal Of Psychiatry | 7 |
| 1998-2002 | Journal Of Clinical Psychiatry | 30 |
|  | Depression And Anxiety | 15 |
|  | International Clinical Psychopharmacology | 14 |
|  | Journal Of Psychopharmacology | 13 |
|  | Journal Of Clinical Psychopharmacology | 12 |
|  | European Journal Of Pharmacology | 10 |
|  | Human Psychopharmacology Clinical And Experimental | 10 |
|  | Cns Drugs | 9 |
|  | Psychopharmacology | 9 |
|  | Annals Of Pharmacotherapy | 8 |
|  | British Journal Of Clinical Pharmacology | 6 |
|  | Journal Of Affective Disorders | 6 |
|  | Neuropharmacology | 6 |
|  | Pharmacology Biochemistry And Behavior | 6 |
|  | Reviews In Contemporary Pharmacotherapy | 6 |
|  | European Neuropsychopharmacology | 5 |
|  | International Journal Of Neuropsychopharmacology | 5 |
|  | Journal Of Clinical Pharmacology | 5 |
|  | Journal Of Pharmacology And Experimental Therapeutics | 5 |
|  | Journal Of Psychiatry Neuroscience | 5 |
| 1993-1997 | International Clinical Psychopharmacology | 10 |
|  | Journal Of Clinical Psychiatry | 9 |
|  | Journal Of Clinical Pharmacology | 5 |
|  | Pharmacotherapy | 5 |
|  | Journal Of Clinical Psychopharmacology | 4 |
|  | Psychopharmacology | 4 |
|  | Psychopharmacology Bulletin | 4 |
|  | Current Therapeutic Research Clinical And Experimental | 3 |
|  | Human Psychopharmacology Clinical And Experimental | 3 |
|  | Journal Of Affective Disorders | 3 |
|  | Journal Of Family Practice | 3 |
|  | American Journal Of Medicine | 2 |
|  | Annals Of Pharmacotherapy | 2 |
|  | British Journal Of Clinical Pharmacology | 2 |
|  | Clinical Therapeutics | 2 |
|  | Cns Drugs | 2 |
|  | Current Opinion In Psychiatry | 2 |
|  | Pharmacopsychiatry | 2 |
|  | Progress In Neuro Psychopharmacology Biological Psychiatry | 2 |
|  | Xenobiotica | 2 |
| 1988-1992 | Drug Development Research | 2 |
|  | British Journal Of Clinical Pharmacology | 1 |
|  | Chirality | 1 |
|  | Journal Of Clinical Pharmacology | 1 |
|  | Journal Of Clinical Psychopharmacology | 1 |

| Fluvoxamine | |  |
| --- | --- | --- |
| **Year** | **Journal** | **No of articles** |
| 2013-2017 | Neuropsychiatric Disease And Treatment | 7 |
|  | European Journal Of Pharmacology | 6 |
|  | Journal Of Clinical Psychopharmacology | 6 |
|  | Psychopharmacology | 6 |
|  | Journal Of Affective Disorders | 5 |
|  | Journal Of Pharmacological Sciences | 5 |
|  | International Journal Of Neuropsychopharmacology | 4 |
|  | Journal Of Clinical Psychiatry | 4 |
|  | Psychiatry Research | 4 |
|  | Annals Of General Psychiatry | 3 |
|  | Biological Pharmaceutical Bulletin | 3 |
|  | Cns Drugs | 3 |
|  | Cochrane Database Of Systematic Reviews | 3 |
|  | Depression And Anxiety | 3 |
|  | European Neuropsychopharmacology | 3 |
|  | Human Psychopharmacology Clinical And Experimental | 3 |
|  | Pharmacology Biochemistry And Behavior | 3 |
|  | Therapeutic Drug Monitoring | 3 |
|  | Annals Of Pharmacotherapy | 2 |
|  | Behavioural Brain Research | 2 |
| 2008-2012 | Human Psychopharmacology Clinical And Experimental | 11 |
|  | Journal Of Psychopharmacology | 11 |
|  | European Journal Of Pharmacology | 9 |
|  | Journal Of Affective Disorders | 9 |
|  | Annals Of General Psychiatry | 7 |
|  | Journal Of Clinical Psychiatry | 7 |
|  | Journal Of Clinical Psychopharmacology | 7 |
|  | Psychopharmacology | 7 |
|  | International Journal Of Neuropsychopharmacology | 6 |
|  | Progress In Neuro Psychopharmacology Biological Psychiatry | 6 |
|  | Behavioural Brain Research | 5 |
|  | Biological Pharmaceutical Bulletin | 5 |
|  | Cochrane Database Of Systematic Reviews | 5 |
|  | Current Neuropharmacology | 5 |
|  | Psychiatry Research | 5 |
|  | Behavioural Pharmacology | 4 |
|  | European Journal Of Clinical Pharmacology | 4 |
|  | Neuropsychiatric Disease And Treatment | 4 |
|  | Pharmacological Reports | 4 |
|  | Psychiatry And Clinical Neurosciences | 4 |
| 2003-2007 | Journal Of Clinical Psychiatry | 22 |
|  | Psychopharmacology | 19 |
|  | Journal Of Clinical Psychopharmacology | 14 |
|  | Progress In Neuro Psychopharmacology Biological Psychiatry | 13 |
|  | Neuropsychopharmacology | 12 |
|  | Human Psychopharmacology Clinical And Experimental | 10 |
|  | European Journal Of Pharmacology | 9 |
|  | European Neuropsychopharmacology | 9 |
|  | International Clinical Psychopharmacology | 8 |
|  | Journal Of Psychopharmacology | 8 |
|  | Therapeutic Drug Monitoring | 8 |
|  | Annals Of Pharmacotherapy | 7 |
|  | International Journal Of Neuropsychopharmacology | 7 |
|  | Journal Of Child And Adolescent Psychopharmacology | 7 |
|  | Neuropsychobiology | 7 |
|  | Pharmacopsychiatry | 7 |
|  | Psychiatry Research | 7 |
|  | Biological Psychiatry | 6 |
|  | Journal Of Affective Disorders | 6 |
|  | Pharmacogenomics Journal | 6 |
| 1998-2002 | Journal Of Clinical Psychopharmacology | 35 |
|  | International Clinical Psychopharmacology | 23 |
|  | Psychopharmacology | 21 |
|  | Journal Of Clinical Psychiatry | 18 |
|  | European Neuropsychopharmacology | 17 |
|  | Human Psychopharmacology Clinical And Experimental | 11 |
|  | Journal Of Psychopharmacology | 11 |
|  | Annals Of Pharmacotherapy | 10 |
|  | Psychiatry Research | 10 |
|  | Therapeutic Drug Monitoring | 10 |
|  | British Journal Of Psychiatry | 9 |
|  | Cns Drugs | 9 |
|  | European Journal Of Pharmacology | 9 |
|  | American Journal Of Psychiatry | 8 |
|  | Biological Psychiatry | 8 |
|  | Journal Of Affective Disorders | 8 |
|  | Pharmacopsychiatry | 8 |
|  | European Journal Of Clinical Pharmacology | 7 |
|  | Acta Psychiatrica Scandinavica | 6 |
|  | International Journal Of Neuropsychopharmacology | 6 |
| 1993-1997 | Human Psychopharmacology Clinical And Experimental | 23 |
|  | Journal Of Clinical Psychopharmacology | 21 |
|  | Psychopharmacology | 21 |
|  | International Clinical Psychopharmacology | 18 |
|  | Journal Of Clinical Psychiatry | 17 |
|  | Acta Psychiatrica Scandinavica | 9 |
|  | Cns Drugs | 9 |
|  | Journal Of Psychopharmacology | 9 |
|  | Pharmacopsychiatry | 9 |
|  | European Journal Of Pharmacology | 8 |
|  | European Neuropsychopharmacology | 8 |
|  | British Journal Of Clinical Pharmacology | 7 |
|  | British Journal Of Psychiatry | 7 |
|  | Clinical Pharmacokinetics | 7 |
|  | Pharmacology Biochemistry And Behavior | 7 |
|  | Journal Of Affective Disorders | 6 |
|  | Therapeutic Drug Monitoring | 6 |
|  | Archives Of General Psychiatry | 5 |
|  | Nordic Journal Of Psychiatry | 5 |
|  | American Journal Of Psychiatry | 4 |
| 1988-1992 | International Clinical Psychopharmacology | 8 |
|  | Journal Of Clinical Psychiatry | 7 |
|  | Human Psychopharmacology Clinical And Experimental | 6 |
|  | Acta Psychiatrica Scandinavica | 4 |
|  | Current Therapeutic Research Clinical And Experimental | 4 |
|  | Journal Of Clinical Psychopharmacology | 4 |
|  | Journal Of Psychiatry Neuroscience | 4 |
|  | British Journal Of Clinical Practice | 3 |
|  | Drugs | 3 |
|  | Journal Of Affective Disorders | 3 |
|  | Pharmacopsychiatry | 3 |
|  | Archives Of General Psychiatry | 2 |
|  | British Journal Of Pharmacology | 2 |
|  | Current Medical Research And Opinion | 2 |
|  | Psychiatric Clinics Of North America | 2 |
|  | Psychopharmacology | 2 |
|  | Therapeutic Drug Monitoring | 2 |
|  | Biological Psychiatry | 1 |
|  | Biopharmaceutics Drug Disposition | 1 |
|  | British Journal Of Clinical Pharmacology | 1 |

| **Clomipramine** |  |  |
| --- | --- | --- |
| **Year** | **Journal** | **No of articles** |
| 2013-2017 | Cochrane Database Of Systematic Reviews | 5 |
|  | Journal Of Affective Disorders | 5 |
|  | Journal Of Psychiatric Research | 5 |
|  | Psychiatric Annals | 5 |
|  | Depression And Anxiety | 3 |
|  | Human Psychopharmacology Clinical And Experimental | 3 |
|  | Indian Journal Of Psychiatry | 3 |
|  | International Clinical Psychopharmacology | 3 |
|  | International Journal Of Neuropsychopharmacology | 3 |
|  | Journal Of Pharmaceutical And Biomedical Analysis | 3 |
|  | Psychiatry Research | 3 |
|  | Psychopharmacology | 3 |
|  | Acta Psychiatrica Scandinavica | 2 |
|  | Anadolu Psikiyatri Dergisi Anatolian Journal Of Psychiatry | 2 |
|  | Annals Of General Psychiatry | 2 |
|  | Bmc Psychiatry | 2 |
|  | Bmj Open | 2 |
|  | Brain Research | 2 |
|  | Epilepsy Behavior | 2 |
|  | European Neuropsychopharmacology | 2 |
| 2008-2012 | Journal Of Psychopharmacology | 15 |
|  | European Neuropsychopharmacology | 7 |
|  | Cochrane Database Of Systematic Reviews | 6 |
|  | Behavioural Brain Research | 5 |
|  | Cns Spectrums | 5 |
|  | Journal Of Clinical Psychopharmacology | 5 |
|  | Therapeutic Drug Monitoring | 5 |
|  | Journal Of Psychiatric Research | 4 |
|  | Neuropsychiatric Disease And Treatment | 4 |
|  | Neuroscience | 4 |
|  | Psychopharmacology | 4 |
|  | Sleep Medicine | 4 |
|  | Acta Psychiatrica Scandinavica | 3 |
|  | Biological Psychiatry | 3 |
|  | Human Psychopharmacology Clinical And Experimental | 3 |
|  | International Clinical Psychopharmacology | 3 |
|  | Journal Of Affective Disorders | 3 |
|  | Journal Of Clinical Psychiatry | 3 |
|  | Journal Of Pharmaceutical And Biomedical Analysis | 3 |
|  | Neurochemical Research | 3 |
| 2003-2007 | Journal Of Clinical Psychiatry | 11 |
|  | Psychopharmacology | 8 |
|  | Progress In Neuro Psychopharmacology Biological Psychiatry | 7 |
|  | Acta Psychiatrica Scandinavica | 6 |
|  | International Clinical Psychopharmacology | 6 |
|  | International Journal Of Neuropsychopharmacology | 6 |
|  | Pharmacopsychiatry | 6 |
|  | Biological Psychiatry | 5 |
|  | Brain Research | 5 |
|  | Cns Drugs | 5 |
|  | European Journal Of Pharmacology | 5 |
|  | Human Psychopharmacology Clinical And Experimental | 5 |
|  | Neuropsychopharmacology | 5 |
|  | Cns Spectrums | 4 |
|  | Depression And Anxiety | 4 |
|  | European Neuropsychopharmacology | 4 |
|  | International Journal Of Impotence Research | 4 |
|  | Journal Of Affective Disorders | 4 |
|  | Journal Of Pharmaceutical And Biomedical Analysis | 4 |
|  | Journal Of Pharmacological Sciences | 4 |
| 1998-2002 | Journal Of Clinical Psychopharmacology | 18 |
|  | Psychopharmacology | 17 |
|  | Journal Of Clinical Psychiatry | 14 |
|  | Biological Psychiatry | 12 |
|  | International Clinical Psychopharmacology | 12 |
|  | Pharmacology Biochemistry And Behavior | 10 |
|  | European Journal Of Pharmacology | 9 |
|  | European Neuropsychopharmacology | 9 |
|  | Human Psychopharmacology Clinical And Experimental | 9 |
|  | Journal Of The American Academy Of Child And Adolescent Psychiatry | 9 |
|  | Cns Drugs | 7 |
|  | Psychiatry Research | 7 |
|  | Archives Of General Psychiatry | 6 |
|  | British Journal Of Psychiatry | 6 |
|  | Journal Of Affective Disorders | 6 |
|  | Neuropsychopharmacology | 6 |
|  | Progress In Neuro Psychopharmacology Biological Psychiatry | 6 |
|  | Therapeutic Drug Monitoring | 6 |
|  | Acta Psychiatrica Scandinavica | 5 |
|  | Journal Of Psychopharmacology | 5 |
| 1993-1997 | Journal Of Affective Disorders | 17 |
|  | Journal Of Clinical Psychopharmacology | 16 |
|  | International Clinical Psychopharmacology | 15 |
|  | Biological Psychiatry | 14 |
|  | Cns Drugs | 14 |
|  | Acta Psychiatrica Scandinavica | 13 |
|  | European Journal Of Pharmacology | 13 |
|  | Progress In Neuro Psychopharmacology Biological Psychiatry | 12 |
|  | Journal Of Clinical Psychiatry | 11 |
|  | Psychopharmacology | 11 |
|  | Pharmacology Biochemistry And Behavior | 9 |
|  | Journal Of Psychopharmacology | 8 |
|  | Journal Of The American Academy Of Child And Adolescent Psychiatry | 8 |
|  | Neuropsychobiology | 8 |
|  | Neuropsychopharmacology | 8 |
|  | Archives Of General Psychiatry | 6 |
|  | European Neuropsychopharmacology | 6 |
|  | European Psychiatry | 6 |
|  | Pharmacopsychiatry | 6 |
|  | Therapeutic Drug Monitoring | 6 |
| 1988-1993 | Acta Psychiatrica Scandinavica | 12 |
|  | Biological Psychiatry | 10 |
|  | Journal Of Clinical Psychiatry | 9 |
|  | Journal Of Clinical Psychopharmacology | 9 |
|  | Psychiatry Research | 9 |
|  | Archives Of General Psychiatry | 8 |
|  | Human Psychopharmacology Clinical And Experimental | 8 |
|  | Journal Of Affective Disorders | 8 |
|  | American Journal Of Psychiatry | 6 |
|  | British Journal Of Psychiatry | 5 |
|  | Canadian Journal Of Psychiatry Revue Canadienne De Psychiatrie | 4 |
|  | Drugs | 4 |
|  | International Clinical Psychopharmacology | 4 |
|  | Pharmacology Biochemistry And Behavior | 4 |
|  | Pharmacopsychiatry | 4 |
|  | Journal Of Pharmacology And Experimental Therapeutics | 3 |
|  | Journal Of The American Academy Of Child And Adolescent Psychiatry | 3 |
|  | Psychiatric Clinics Of North America | 3 |
|  | Psychopharmacology | 3 |
|  | Psychopharmacology Bulletin | 3 |

| **ECT** |  |  |
| --- | --- | --- |
| **Year** | **Journal** | **No of articles** |
| 2013-2017 | Journal Of ECT | 167 |
|  | Journal Of Affective Disorders | 46 |
|  | Journal Of Psychiatric Research | 21 |
|  | Neuropsychiatric Disease And Treatment | 21 |
|  | Brain Stimulation | 18 |
|  | International Journal Of Neuropsychopharmacology | 17 |
|  | Journal Of Clinical Psychiatry | 17 |
|  | European Neuropsychopharmacology | 16 |
|  | Psychiatry Research | 16 |
|  | Translational Psychiatry | 13 |
|  | World Journal Of Biological Psychiatry | 13 |
|  | Acta Psychiatrica Scandinavica | 11 |
|  | European Archives Of Psychiatry And Clinical Neuroscience | 11 |
|  | Progress In Neuro Psychopharmacology Biological Psychiatry | 11 |
|  | Journal Of Neural Transmission | 10 |
|  | American Journal Of Psychiatry | 9 |
|  | Bmc Psychiatry | 9 |
|  | Current Psychiatry Reports | 9 |
|  | Psychiatry Research Neuroimaging | 9 |
|  | Indian Journal Of Psychiatry | 8 |
| 2008-2012 | Journal Of ECT | 187 |
|  | Journal Of Affective Disorders | 53 |
|  | Brain Stimulation | 19 |
|  | Journal Of Clinical Psychiatry | 16 |
|  | Progress In Neuro Psychopharmacology Biological Psychiatry | 16 |
|  | Psychiatry Research | 14 |
|  | World Journal Of Biological Psychiatry | 14 |
|  | Journal Of Psychiatric Research | 13 |
|  | Biological Psychiatry | 12 |
|  | Neuroscience Letters | 12 |
|  | International Journal Of Neuropsychopharmacology | 10 |
|  | Medical Hypotheses | 10 |
|  | Australian And New Zealand Journal Of Psychiatry | 7 |
|  | Bipolar Disorders | 7 |
|  | Neuropsychopharmacology | 7 |
|  | Pharmacopsychiatry | 7 |
|  | Bmc Psychiatry | 6 |
|  | British Journal Of Psychiatry | 6 |
|  | Journal Of Anesthesia | 6 |
|  | Neuropsychobiology | 6 |
| 2003-2007 | Journal Of ECT | 140 |
|  | Journal Of Clinical Psychiatry | 22 |
|  | Biological Psychiatry | 21 |
|  | Journal Of Affective Disorders | 17 |
|  | CNS Spectrums | 12 |
|  | Neuropsychopharmacology | 11 |
|  | Progress In Neuro Psychopharmacology Biological Psychiatry | 10 |
|  | Australian And New Zealand Journal Of Psychiatry | 9 |
|  | Psychiatric Annals | 9 |
|  | Psychiatry Research Neuroimaging | 9 |
|  | American Journal Of Psychiatry | 8 |
|  | International Journal Of Neuropsychopharmacology | 8 |
|  | World Journal Of Biological Psychiatry | 8 |
|  | British Journal Of Psychiatry | 7 |
|  | Psychiatry Research | 7 |
|  | Bipolar Disorders | 6 |
|  | International Journal Of Psychiatry In Clinical Practice | 6 |
|  | Journal Of Psychiatric Research | 6 |
|  | Clinical Neurophysiology | 5 |
|  | Depression And Anxiety | 5 |
| 1998-2002 | Journal Of ECT | 110 |
|  | Biological Psychiatry | 22 |
|  | Journal Of Affective Disorders | 21 |
|  | Journal Of Clinical Psychiatry | 16 |
|  | Progress In Neuro Psychopharmacology Biological Psychiatry | 10 |
|  | Psychiatry And Clinical Neurosciences | 10 |
|  | Acta Psychiatrica Scandinavica | 8 |
|  | American Journal Of Geriatric Psychiatry | 8 |
|  | American Journal Of Psychiatry | 8 |
|  | International Journal Of Geriatric Psychiatry | 8 |
|  | Cns Drugs | 7 |
|  | Depression And Anxiety | 7 |
|  | International Journal Of Neuropsychopharmacology | 7 |
|  | Neuropsychopharmacology | 7 |
|  | Psychiatric Annals | 7 |
|  | Archives Of General Psychiatry | 5 |
|  | Australian And New Zealand Journal Of Psychiatry | 5 |
|  | Canadian Journal Of Psychiatry Revue Canadienne De Psychiatrie | 5 |
|  | Psychiatric Clinics Of North America | 5 |
|  | Psychiatry Research | 5 |
| 1993-1997 | Convulsive Therapy | 53 |
|  | Biological Psychiatry | 24 |
|  | American Journal Of Psychiatry | 17 |
|  | Journal Of Affective Disorders | 15 |
|  | International Journal Of Geriatric Psychiatry | 12 |
|  | CNS Drugs | 11 |
|  | Psychopharmacology Bulletin | 11 |
|  | Australian And New Zealand Journal Of Psychiatry | 10 |
|  | Journal Of Neuropsychiatry And Clinical Neurosciences | 9 |
|  | Journal Of Clinical Psychiatry | 8 |
|  | Psychiatric Annals | 8 |
|  | British Journal Of Psychiatry | 7 |
|  | Canadian Journal Of Psychiatry Revue Canadienne De Psychiatrie | 7 |
|  | Psychiatric Clinics Of North America | 7 |
|  | Acta Psychiatrica Scandinavica | 6 |
|  | American Journal Of Geriatric Psychiatry | 5 |
|  | Human Psychopharmacology Clinical And Experimental | 5 |
|  | Journal Of Psychiatric Research | 5 |
|  | Progress In Neuro Psychopharmacology Biological Psychiatry | 5 |
|  | Psychological Medicine | 5 |
| 1988-1992 | Convulsive Therapy | 43 |
|  | Journal Of Clinical Psychiatry | 11 |
|  | British Journal Of Psychiatry | 10 |
|  | American Journal Of Psychiatry | 8 |
|  | Psychiatric Clinics Of North America | 8 |
|  | International Journal Of Geriatric Psychiatry | 7 |
|  | Biological Psychiatry | 6 |
|  | Journal Of Affective Disorders | 6 |
|  | Psychiatry Research | 6 |
|  | Acta Psychiatrica Scandinavica | 5 |
|  | Archives Of General Psychiatry | 5 |
|  | Canadian Journal Of Psychiatry Revue Canadienne De Psychiatrie | 5 |
|  | Psychopharmacology Bulletin | 5 |
|  | Journal Of Neuropsychiatry And Clinical Neurosciences | 4 |
|  | Progress In Neuro Psychopharmacology Biological Psychiatry | 4 |
|  | International Clinical Psychopharmacology | 3 |
|  | Journal Of Nervous And Mental Disease | 3 |
|  | Psychosomatics | 3 |
|  | American Family Physician | 2 |
|  | Australian And New Zealand Journal Of Psychiatry | 2 |

| **rTMS** |  |  |
| --- | --- | --- |
| **Year** | **Journal** | **No of articles** |
| 2013-2017 | Brain Stimulation | 49 |
|  | Journal Of Affective Disorders | 21 |
|  | Journal Of ECT | 18 |
|  | Clinical Neurophysiology | 13 |
|  | Psychiatry Research | 13 |
|  | Frontiers In Human Neuroscience | 12 |
|  | Journal Of Psychiatric Research | 10 |
|  | Journal Of Neural Transmission | 8 |
|  | Neuroimage | 8 |
|  | Neuroscience Letters | 7 |
|  | Clinical Psychopharmacology And Neuroscience | 6 |
|  | Depression And Anxiety | 6 |
|  | Journal Of Neuropsychiatry And Clinical Neurosciences | 6 |
|  | Neuroscience And Biobehavioral Reviews | 6 |
|  | Psychological Medicine | 6 |
|  | Restorative Neurology And Neuroscience | 6 |
|  | Trials | 6 |
|  | Biological Psychiatry | 5 |
|  | Cerebral Cortex | 5 |
|  | Cochrane Database Of Systematic Reviews | 5 |
| 2008-2012 | Brain Stimulation | 36 |
|  | Journal Of Affective Disorders | 18 |
|  | Journal Of Ect | 16 |
|  | Clinical Neurophysiology | 15 |
|  | World Journal Of Biological Psychiatry | 11 |
|  | Depression And Anxiety | 9 |
|  | International Journal Of Neuropsychopharmacology | 9 |
|  | Clinical Eeg And Neuroscience | 8 |
|  | Journal Of Clinical Psychiatry | 8 |
|  | Neuropsychobiology | 7 |
|  | Psychological Medicine | 7 |
|  | Experimental Brain Research | 6 |
|  | Progress In Neuro Psychopharmacology Biological Psychiatry | 6 |
|  | Psychiatry Research | 6 |
|  | Cerebral Cortex | 5 |
|  | European Journal Of Neuroscience | 5 |
|  | Experimental Neurology | 5 |
|  | Journal Of Physiology London | 5 |
|  | Neuroscience Letters | 5 |
|  | Pain | 5 |
| 2003-2007 | Clinical Neurophysiology | 17 |
|  | International Journal Of Neuropsychopharmacology | 10 |
|  | Journal Of Affective Disorders | 10 |
|  | Neuroscience Letters | 10 |
|  | Journal Of Psychiatric Research | 9 |
|  | Biological Psychiatry | 8 |
|  | Experimental Brain Research | 8 |
|  | Psychiatry Research | 8 |
|  | Journal Of Clinical Psychiatry | 7 |
|  | Neuroreport | 7 |
|  | Journal Of Neurology Neurosurgery And Psychiatry | 6 |
|  | Depression And Anxiety | 5 |
|  | Progress In Neuro Psychopharmacology Biological Psychiatry | 5 |
|  | Psychological Medicine | 5 |
|  | American Journal Of Psychiatry | 4 |
|  | British Journal Of Psychiatry | 4 |
|  | Journal Of Ect | 4 |
|  | Journal Of Neuropsychiatry And Clinical Neurosciences | 4 |
|  | Journal Of Psychiatry Neuroscience | 4 |
|  | Neuroendocrinology Letters | 4 |
| 1998-2002 | Biological Psychiatry | 15 |
|  | Clinical Neurophysiology | 8 |
|  | Experimental Brain Research | 7 |
|  | Neuroreport | 6 |
|  | Depression And Anxiety | 5 |
|  | Human Psychopharmacology Clinical And Experimental | 5 |
|  | Journal Of Ect | 5 |
|  | Journal Of Neuropsychiatry And Clinical Neurosciences | 5 |
|  | Neurology | 5 |
|  | Neuropsychopharmacology | 5 |
|  | Neuroscience Letters | 5 |
|  | Brain Research | 4 |
|  | International Journal Of Neuropsychopharmacology | 4 |
|  | Psychiatry Research Neuroimaging | 4 |
|  | European Journal Of Neuroscience | 3 |
|  | Journal Of Psychiatric Research | 3 |
|  | Journal Of The Neurological Sciences | 3 |
|  | Muscle Nerve | 3 |
|  | Neuropsychiatry Neuropsychology And Behavioral Neurology | 3 |
|  | Progress In Neuro Psychopharmacology Biological Psychiatry | 3 |
| 1993-1997 | Neuroreport | 2 |
|  | Australian And New Zealand Journal Of Psychiatry | 1 |
|  | Convulsive Therapy | 1 |
|  | Development And Psychopathology | 1 |
|  | Journal Of Neuropsychiatry And Clinical Neurosciences | 1 |
|  | Lancet | 1 |

| **VNS** |  |  |
| --- | --- | --- |
| **Year** | **Journal** | **No of articles** |
| 2013-2017 | Brain Stimulation | 11 |
|  | Neuroscience And Biobehavioral Reviews | 6 |
|  | Headache | 5 |
|  | Journal Of ECT | 5 |
|  | Epilepsy Behavior | 4 |
|  | Journal Of Affective Disorders | 4 |
|  | Journal Of Neural Transmission | 4 |
|  | Biological Psychiatry | 3 |
|  | Current Geriatrics Reports | 3 |
|  | Evidence Based Complementary And Alternative Medicine | 3 |
|  | Journal Of Neurochemistry | 3 |
|  | Neurotherapeutics | 3 |
|  | Pain | 3 |
|  | Seizure European Journal Of Epilepsy | 3 |
|  | World Journal Of Biological Psychiatry | 3 |
|  | American Journal Of Geriatric Psychiatry | 2 |
|  | Bipolar Disorders | 2 |
|  | Clinical Psychopharmacology And Neuroscience | 2 |
|  | Cochrane Database Of Systematic Reviews | 2 |
|  | Current Neuropharmacology | 2 |
| 2008-2012 | Brain Stimulation | 7 |
|  | Pharmacopsychiatry | 7 |
|  | Epilepsy Behavior | 6 |
|  | Journal Of Ect | 5 |
|  | Neuropsychopharmacology | 5 |
|  | Neurosurgical Focus | 5 |
|  | Experimental Neurology | 4 |
|  | Journal Of Affective Disorders | 4 |
|  | Appetite | 3 |
|  | Biological Psychiatry | 3 |
|  | Current Treatment Options In Neurology | 3 |
|  | International Journal Of Neuropsychopharmacology | 3 |
|  | Medical Hypotheses | 3 |
|  | Neuroscience And Biobehavioral Reviews | 3 |
|  | Neurosurgery | 3 |
|  | World Journal Of Biological Psychiatry | 3 |
|  | Annals Of Medicine | 2 |
|  | Australian And New Zealand Journal Of Psychiatry | 2 |
|  | Bipolar Disorders | 2 |
|  | Clinical Neurology And Neurosurgery | 2 |
| 2003-2017 | Biological Psychiatry | 8 |
|  | Epilepsy Behavior | 8 |
|  | Cns Spectrums | 6 |
|  | Clinical Eeg And Neuroscience | 3 |
|  | Journal Of Affective Disorders | 3 |
|  | Journal Of Neural Transmission | 3 |
|  | Journal Of Psychiatric Research | 3 |
|  | Neuropsychopharmacology | 3 |
|  | Psychiatric Annals | 3 |
|  | Seizure European Journal Of Epilepsy | 3 |
|  | Acta Neurologica Scandinavica | 2 |
|  | Acta Neuropsychiatrica | 2 |
|  | Brain Research | 2 |
|  | Child And Adolescent Psychiatric Clinics Of North America | 2 |
|  | Cns Drugs | 2 |
|  | Current Opinion In Psychiatry | 2 |
|  | International Journal Of Neuropsychopharmacology | 2 |
|  | Journal Of Clinical Psychiatry | 2 |
|  | Journal Of Ect | 2 |
|  | Journal Of Psychosocial Nursing And Mental Health Services | 2 |
| 1998-2002 | Pilepsy Behavior | 6 |
|  | Neurology | 4 |
|  | Biological Psychiatry | 2 |
|  | Clinical Neuroscience Research | 2 |
|  | Epilepsia | 2 |
|  | American Journal Of Physiology Regulatory Integrative And Comparative Physiology | 1 |
|  | British Journal Of Pharmacology | 1 |
|  | Current Opinion In Neurology | 1 |
|  | Epilepsy Research | 1 |
|  | Investigative Radiology | 1 |
|  | Journal Of Clinical Neurophysiology | 1 |
|  | Journal Of Clinical Psychiatry | 1 |
|  | Journal Of Ect | 1 |
|  | Journal Of Psychiatric Research | 1 |
|  | Journal Of Psychosomatic Research | 1 |
|  | Mayo Clinic Proceedings | 1 |
|  | Medical Hypotheses | 1 |
|  | Neuropsychiatry Neuropsychology And Behavioral Neurology | 1 |
|  | Neuropsychopharmacology | 1 |
|  | Neuroscience | 1 |
| 1993-1998 | Canadian Journal Of Neurological Sciences | 1 |
|  | Naunyn Schmiedebergs Archives Of Pharmacology | 1 |
|  | Physiology Behavior | 1 |

| **DBS** |  |  |
| --- | --- | --- |
| **Year** | **Journal** | **No of articles** |
| 2013-2017 | Neurosciences | 293 |
|  | Clinical Neurology | 183 |
|  | Psychiatry | 181 |
|  | Pharmacology Pharmacy | 62 |
|  | Surgery | 57 |
|  | Behavioral Sciences | 46 |
|  | Neuroimaging | 38 |
|  | Psychology | 33 |
|  | Psychology Clinical | 25 |
|  | Medicine Research Experimental | 21 |
|  | Radiology Nuclear Medicine Medical Imaging | 17 |
|  | Medicine General Internal | 14 |
|  | Biochemistry Molecular Biology | 12 |
|  | Anatomy Morphology | 8 |
|  | Geriatrics Gerontology | 7 |
|  | Psychology Experimental | 7 |
|  | Health Care Sciences Services | 5 |
|  | Physiology | 5 |
|  | Ethics | 4 |
|  | Medical Ethics | 4 |
| 2008-2012 | Neurosciences | 165 |
|  | Psychiatry | 121 |
|  | Clinical Neurology | 111 |
|  | Surgery | 45 |
|  | Pharmacology Pharmacy | 32 |
|  | Behavioral Sciences | 23 |
|  | Neuroimaging | 23 |
|  | Radiology Nuclear Medicine Medical Imaging | 19 |
|  | Biochemistry Molecular Biology | 12 |
|  | Medicine General Internal | 9 |
|  | Medicine Research Experimental | 9 |
|  | Psychology | 8 |
|  | Psychology Clinical | 8 |
|  | Cell Biology | 7 |
|  | Ethics | 6 |
|  | Social Sciences Biomedical | 6 |
|  | Medical Ethics | 4 |
|  | Anatomy Morphology | 3 |
|  | Psychology Experimental | 3 |
|  | Social Issues | 3 |
| 2003-2007 | Clinical Neurology | 25 |
|  | Psychiatry | 24 |
|  | Neurosciences | 19 |
|  | Surgery | 10 |
|  | Pharmacology Pharmacy | 5 |
|  | Physiology | 3 |
|  | Anesthesiology | 2 |
|  | Behavioral Sciences | 2 |
|  | Dentistry Oral Surgery Medicine | 1 |
|  | Medicine Research Experimental | 1 |
|  | Psychology | 1 |
|  | Psychology Clinical | 1 |
|  | Radiology Nuclear Medicine Medical Imaging | 1 |
|  | Rehabilitation | 1 |
| 1998-2002 | Clinical Neurology | 3 |
|  | Behavioral Sciences | 1 |
|  | Biotechnology Applied Microbiology | 1 |
|  | Medicine General Internal | 1 |
|  | Neurosciences | 1 |
|  | Psychiatry | 1 |
|  | Psychology Experimental | 1 |
|  | Toxicology | 1 |
| 1993-1997 | Neurosciences | 1 |
|  | Psychology | 1 |

| **tDCS** |  | |  | |
| --- | --- | --- | --- | --- |
| **Year** | **Journal** | | **No of articles** | |
| 2013-2017 | Brain Stimulation | 23 | |  |
|  | Journal Of Affective Disorders | 16 | |  |
|  | International Journal Of Neuropsychopharmacology | 9 | |  |
|  | Clinical Neurophysiology | 8 | |  |
|  | Neuropsychologia | 7 | |  |
|  | Trials | 7 | |  |
|  | Frontiers In Psychiatry | 6 | |  |
|  | Neuroscience And Biobehavioral Reviews | 6 | |  |
|  | Frontiers In Human Neuroscience | 5 | |  |
|  | Journal Of ECT | 5 | |  |
|  | Neuroimage | 5 | |  |
|  | Neuropsychiatric Disease And Treatment | 5 | |  |
|  | Neuropsychopharmacology | 5 | |  |
|  | Neuroscience Letters | 5 | |  |
|  | Progress In Neuro Psychopharmacology Biological Psychiatry | 5 | |  |
|  | Restorative Neurology And Neuroscience | 5 | |  |
|  | Journal Of Physiology London | 4 | |  |
|  | Neural Plasticity | 4 | |  |
|  | Neuromodulation | 4 | |  |
|  | Biological Psychiatry | 3 | |  |
| 2008-2012 | Brain Stimulation | 11 | |  |
|  | Clinical Neurophysiology | 4 | |  |
|  | Journal Of Affective Disorders | 4 | |  |
|  | Journal Of ECT | 4 | |  |
|  | Neuropsychopharmacology | 4 | |  |
|  | Neuroscience Letters | 4 | |  |
|  | European Journal Of Neuroscience | 3 | |  |
|  | Experimental Brain Research | 3 | |  |
|  | International Journal Of Neuropsychopharmacology | 3 | |  |
|  | Journal Of Neuroscience | 3 | |  |
|  | Journal Of Physiology London | 3 | |  |
|  | Neuroimage | 3 | |  |
|  | Restorative Neurology And Neuroscience | 3 | |  |
|  | Biological Psychiatry | 2 | |  |
|  | BMC Neuroscience | 2 | |  |
|  | British Journal Of Psychiatry | 2 | |  |
|  | Cerebral Cortex | 2 | |  |
|  | Current Opinion In Psychiatry | 2 | |  |
|  | Journal Of Neurology Neurosurgery And Psychiatry | 2 | |  |
|  | Journal Of Neurophysiology | 2 | |  |
| 2003-2007 | Arthritis And Rheumatism | 1 | |  |
|  | Brain Research Bulletin | 1 | |  |
|  | Brain Research Reviews | 1 | |  |
|  | Current Opinion In Neurology | 1 | |  |
|  | Current Psychiatry Reviews | 1 | |  |
|  | Experimental Brain Research | 1 | |  |
|  | Experimental Neurology | 1 | |  |
|  | Journal Of Affective Disorders | 1 | |  |
|  | Journal Of Neuroscience | 1 | |  |
|  | Journal Of The Neurological Sciences | 1 | |  |
|  | Neuroimage | 1 | |  |
|  | Neuroscience Letters | 1 | |  |
|  | Pain | 1 | |  |
|  | Restorative Neurology And Neuroscience | 1 | |  |
| 1998-2002 | Bipolar Disorders | 1 | |  |
|  | Journal Of Physiology London | 1 | |  |
