# Supplementary material for: Indices of Change, Expectations, and Popularity of Biological Treatments for Major Depressive Disorder between 1988 and 2017: A Scientometric Analysis
Source: Int J Environ Res Public Health. 2019 Jun 26;16(13):2255. doi: 10.3390/ijerph16132255 (PMC6651662; doi:10.3390/ijerph16132255)
Supplement: Supplementary file 1 [file ijerph-16-02255-s001.zip › IJERPH Supplementary files/9. Supplementary 1 Search Strategy.docx]

**Supplementary file 1. The search strategy**

| **No** | **Search query** | **Result** |
| --- | --- | --- |
| #1 | TS=(Depress* OR antidepressant OR tricyclic-antidepressant OR TCA OR selective serotonin reuptake inhibitor OR SSRI OR serotonin noradrenaline reuptake inhibitor OR serotonin norepinephrine reuptake inhibitor, SNRI OR monoamine oxidase inhibitor OR MAOI OR noradrenaline/norepinephrine-dopamine reuptake inhibitor OR NDRI OR noradrenergic specific serotonergic antidepressant OR NASSA OR serotonin antagonist and reuptake inhibitor OR SARI OR electroconvulsive therapy OR ECT OR repetitive transcranial magnetic stimulation or rTMS OR vagus nerve stimulation OR VNS OR deep brain stimulation OR DBS OR transcranial direct current stimulation OR tDCS) | 598,317 |
| #2 | SU=(Allergy OR Anatomy & Morphology OR Anesthesiology OR Anthropology OR Behavioral Sciences OR Biochemistry & Molecular Biology OR Biophysics OR Biotechnology OR Applied Microbiology OR Cardiovascular System OR Cardiology OR Cell Biology OR Critical Care Medicine OR Dentistry, Oral Surgery & Medicine OR Dermatology OR Emergency Medicine OR Endocrinology & Metabolism OR Evolutionary Biology OR Gastroenterology & Hepatology OR General & Internal Medicine OR Genetics & Heredity OR Geriatrics & Gerontology OR Health Care Sciences &Services OR Hematology OR Immunology OR Infectious Diseases OR Integrative & Complementary Medicine OR Legal Medicine OR Life Sciences Biomedicine Other Topics OR Medical Ethics OR Medical Informatics OR Medical Laboratory Technology OR Microbiology OR Mycology OR Neurosciences & Neurology OR Nursing OR Nutrition & Dietetics OR Obstetrics & Gynecology OR Oncology OR Ophthalmology OR Orthopedics OR Otorhinolaryngology OR Pathology OR Pediatrics OR Pharmacology & Pharmacy OR Physiology OR Psychiatry OR Public, Environmental & Occupational Health OR Radiology, Nuclear Medicine & Medical Imaging OR Rehabilitation OR Reproductive Biology OR Research & Experimental Medicine OR Respiratory System OR Rheumatology OR Sport Sciences OR Substance Abuse OR Surgery OR Toxicology OR Transplantation OR Tropical Medicine OR Urology & Nephrology OR Biochemistry & Molecular Biology OR Mathematical & Computational Biology OR Public, Environmental & Occupational Health Chemistry OR Social Issues OR Social Sciences Other Topics OR Social Work OR Sociology OR Women's Studies OR Biomedical Social Sciences OR Psychology) | 30,979,429 |
| #3 | SU=(Biodiversity & Conservation OR Entomology OR Evolutionary-Biology OR Fisheries OR Food Science & Technology OR Forestry OR Marine & Freshwater Biology OR Paleontology OR Parasitology OR Plant Sciences OR Veterinary Sciences OR Virology OR Zoology OR Developmental Biology OR Astronomy & Astrophysics OR Crystallography OR Electrochemistry OR Geochemistry & Geophysics OR Geology OR Mathematics OR Meteorology & Atmospheric Sciences OR Mineralogy OR Mining & Mineral Processing OR Oceanography OR Optics OR Physical Geography OR Physics OR Polymer Science OR Thermodynamics OR Water Resources OR Acoustics OR Automation & Control Systems OR Computer Science OR Construction & Building Technology OR Energy & Fuels OR Engineering OR Imaging Science & Photographic Technology OR Information Science & Library Science OR Instruments & Instrumentation OR Materials Science OR Mechanics OR Metallurgy & Metallurgical Engineering OR Microscopy OR Nuclear Science & Technology OR Operations Research & Management Science OR Remote Sensing OR Robotics OR Science & Technology Other Topics OR Spectroscopy OR Telecommunications OR Transportation OR Architecture OR Art OR Arts & Humanities Other Topics OR Asian Studies OR Classics OR Dance OR Film, Radio & Television OR History OR History & Philosophy of Science OR Literature OR Music OR Philosophy OR Religion OR Theater OR Archaeology OR Area Studies OR Business & Economics OR Communication OR Criminology & Penology OR Cultural Studies OR Demography OR Education & Educational Research OR Ethnic Studies OR Family Studies OR Geography OR Government & Law OR International Relations OR Linguistics OR Mathematical Methods In Social Sciences OR Public Administration OR Urban Studies) | 39,034,298 |
| # 4 | #2 AND #1 | 514,304 |
| # 5 | #4 NOT #3 | 489,496 |
| # 6 | #4 NOT #3  Refined by: [excluding] PUBLICATION YEARS: ( 2018 ) | 471,033 |
| #7 | #4 NOT #3  Refined by: [excluding] PUBLICATION YEARS: ( 2018 ) AND [excluding] DOCUMENT TYPES: ( MEETING ABSTRACT OR PROCEEDINGS PAPER OR LETTER OR EDITORIAL MATERIAL OR BOOK CHAPTER OR NOTE OR BOOK REVIEW OR CORRECTION OR NEWS ITEM OR DISCUSSION OR REPRINT OR BOOK OR CORRECTION ADDITION OR RETRACTED PUBLICATION OR BIOGRAPHICAL ITEM OR ABSTRACT OF PUBLISHED ITEM OR RETRACTION OR ITEM ABOUT AN INDIVIDUAL OR DATABASE REVIEW OR BIBLIOGRAPHY OR EARLY ACCESS OR SOFTWARE REVIEW OR ART EXHIBIT REVIEW OR FILM REVIEW OR MEETING SUMMARY OR POETRY ) | 377,198 |
| # 8 | #4 NOT #3  Refined by: [excluding] PUBLICATION YEARS: ( 2018 ) AND [excluding] DOCUMENT TYPES: ( MEETING ABSTRACT OR PROCEEDINGS PAPER OR LETTER OR EDITORIAL MATERIAL OR BOOK CHAPTER OR NOTE OR BOOK REVIEW OR CORRECTION OR NEWS ITEM OR DISCUSSION OR REPRINT OR BOOK OR CORRECTION ADDITION OR RETRACTED PUBLICATION OR BIOGRAPHICAL ITEM OR ABSTRACT OF PUBLISHED ITEM OR RETRACTION OR ITEM ABOUT AN INDIVIDUAL OR DATABASE REVIEW OR BIBLIOGRAPHY OR EARLY ACCESS OR SOFTWARE REVIEW OR ART EXHIBIT REVIEW OR FILM REVIEW OR MEETING SUMMARY OR POETRY ) AND [excluding] LANGUAGES: ( GERMAN OR FRENCH OR SPANISH OR RUSSIAN OR TURKISH OR PORTUGUESE OR POLISH OR ITALIAN OR KOREAN OR JAPANESE OR CZECH OR DUTCH OR CHINESE OR HUNGARIAN OR SERBIAN OR CROATIAN OR SLOVENIAN OR GREEK OR DANISH OR ICELANDIC OR NORWEGIAN OR SLOVAK OR LITHUANIAN OR ROMANIAN OR UKRAINIAN OR PERSIAN OR MALAY OR AFRIKAANS OR ESTONIAN OR ARABIC OR SWEDISH OR CATALAN OR WELSH OR BULGARIAN OR GEORGIAN OR SERBO CROATIAN OR GAELIC OR INDONESIAN ) | 355,341 |
| # 9 | #4 NOT #3  Refined by: [excluding] PUBLICATION YEARS: ( 2018 ) AND [excluding] DOCUMENT TYPES: ( MEETING ABSTRACT OR PROCEEDINGS PAPER OR LETTER OR EDITORIAL MATERIAL OR BOOK CHAPTER OR NOTE OR BOOK REVIEW OR CORRECTION OR NEWS ITEM OR DISCUSSION OR REPRINT OR BOOK OR CORRECTION ADDITION OR RETRACTED PUBLICATION OR BIOGRAPHICAL ITEM OR ABSTRACT OF PUBLISHED ITEM OR RETRACTION OR ITEM ABOUT AN INDIVIDUAL OR DATABASE REVIEW OR BIBLIOGRAPHY OR EARLY ACCESS OR SOFTWARE REVIEW OR ART EXHIBIT REVIEW OR FILM REVIEW OR MEETING SUMMARY OR POETRY ) AND [excluding] LANGUAGES: ( GERMAN OR FRENCH OR SPANISH OR RUSSIAN OR TURKISH OR PORTUGUESE OR POLISH OR ITALIAN OR KOREAN OR JAPANESE OR CZECH OR DUTCH OR CHINESE OR HUNGARIAN OR SERBIAN OR CROATIAN OR SLOVENIAN OR GREEK OR DANISH OR ICELANDIC OR NORWEGIAN OR SLOVAK OR LITHUANIAN OR ROMANIAN OR UKRAINIAN OR PERSIAN OR MALAY OR AFRIKAANS OR ESTONIAN OR ARABIC OR SWEDISH OR CATALAN OR WELSH OR BULGARIAN OR GEORGIAN OR SERBO CROATIAN OR GAELIC OR INDONESIAN ) AND [excluding] AUTHORS: ( ANONYMOUS )  Indexes=SCI-EXPANDED, SSCI, A&HCI, CPCI-S, CPCI-SSH, BKCI-S, BKCI-SSH, ESCI, CCR-EXPANDED, IC Timespan=All years | 355,116 |
| # 10 | TS=( levomilnacipran) | 116 |
| # 11 | #10 AND #9 | 58 |
| # 12 | TS=(vilazodone) | 198 |
| # 13 | #12 AND #9 | 115 |
| # 14 | TS=(desvenlafaxine) | 389 |
| # 15 | #14 AND #9 | 182 |
| # 16 | TS=(vortioxetine) | 424 |
| # 17 | #16 AND #9 | 199 |
| # 18 | TS=(agomelatine) | 1,059 |
| # 19 | #18 AND #9 | 448 |
| # 20 | TS=mirtazapine | 2,479 |
| # 21 | #20 AND #9 | 1,268 |
| # 22 | TS=milnacipran | 921 |
| # 23 | #22 AND #9 | 454 |
| # 24 | TS=nefazodone | 907 |
| # 25 | #24 AND #9 | 512 |
| # 26 | TS=moclobemide | 1,209 |
| # 27 | #26 AND #9 | 593 |
| # 28 | TS=reboxetine | 1,107 |
| # 29 | #28 AND #9 | 607 |
| # 30 | TS=trazodone | 1,809 |
| # 31 | #30 AND #9 | 709 |
| # 32 | TS=nortriptyline | 2,411 |
| # 33 | #32 AND #9 | 1,193 |
| # 34 | TS=duloxetine | 3,150 |
| # 35 | #34 AND #9 | 1,312 |
| # 36 | TS=bupropion | 5,523 |
| # 37 | #36 AND #9 | 1,649 |
| # 38 | TS=escitalopram | 3,003 |
| # 39 | #38 AND #9 | 1,603 |
| # 40 | TS=clomipramine | 4,050 |
| # 41 | #40 AND #9 | 1,786 |
| # 42 | TS=fluvoxamine | 3,906 |
| # 43 | #42 AND #9 | 1,944 |
| # 44 | TS=venlafaxine | 5,440 |
| # 45 | #44 AND #9 | 2,849 |
| # 46 | TS=amitriptyline | 7,656 |
| # 47 | #46 AND #9 | 3,177 |
| # 48 | TS=sertraline | 6,087 |
| # 49 | #48 AND #9 | 3,349 |
| # 50 | TS=citalopram | 6,125 |
| # 51 | #50 AND #9 | 3,431 |
| # 52 | TS=paroxetine | 8,052 |
| # 53 | #52 AND #9 | 4,349 |
| # 54 | TS=fluoxetine | 18,008 |
| # 55 | #54 AND #9 | 9,244 |
| # 56 | TS=(ECT OR electroconvulsive-therapy) | 16,124 |
| # 57 | #56 AND #9 | 3,554 |
| # 58 | TS=(rTMS OR repetitive-transcranial-magnetic-stimulation) | 6,169 |
| # 59 | #58 AND #9 | 1,371 |
| # 60 | TS=(VNS OR vagus-nerve-stimulation) | 5,380 |
| # 61 | #60 AND #9 | 552 |
| # 62 | TS=(DBS OR deep-brain-stimulation) | 22,588 |
| # 63 | #62 AND #9 | 1,475 |
| # 64 | TS=(Parkinson) | 110,446 |
| # 65 | #63 NOT #64 | 984 |
| # 66 | TS=(tDCS OR transcranial-direct-current-stimulation) | 4,894 |
| # 67 | #66 AND #9 | 438 |
